# Bacterial Isolates Associated with Mortality Events in Brown Trout (*Salmo trutta)* Restocking Farms in Spain: A Descriptive Field Study

**DOI:** 10.3390/ani15172532

**Published:** 2025-08-28

**Authors:** Augusto Vargas-González, Miguel Barajas, Tania Pérez-Sánchez

**Affiliations:** 1 Biochemistry Area, Health Science Department, Faculty of Health Sciences, Public University of Navarra, 31008 Pamplona, Spain; augusto.vargas@unavarra.es (A.V.-G.); miguel.barajas@unavarra.es (M.B.); 2 Official College of Veterinary Surgeons of Huesca, 22004 Huesca, Spain

**Keywords:** *Salmo trutta*, bacterial isolates, antibiotic resistance, aquaculture, MALDI-TOF MS, qPCR, One Health

## Abstract

This study aimed to identify bacterial isolates associated with mortality events in *Salmo trutta* rearing farms in Spain and to assess their antibiotic resistance profiles. The analysis covered five fish farms: two with a recent history of antibiotic use and three without any antibiotic application in the six months prior to sampling. Tissue samples were collected from moribund fish displaying clinical signs such as erratic swimming, ocular hemorrhages, fin hemorrhages, and skin lesions during disease outbreaks in 2022 and 2023. The samples were analyzed using real-time PCR, amplification and sequencing of the 16S rRNA gene and the ITS-1 intergenic spacer, and MALDI-TOF mass spectrometry. A total of 19 bacterial isolates were identified, with Gram-negative bacteria, particularly *Aeromonas* spp., being the most prevalent. Other identified taxa included *Plesiomonas* sp., *Hafnia alvei*, *Pseudomonas fulva*, and *Kluyvera intermedia*, as well as Gram-positive species such as *Carnobacterium maltaromaticum*, *Lactococcus* sp., and *Enterococcus faecium*. Notably, resistant strains were found in four of the five farms, even in those that had not administered antibiotics, suggesting that environmental contamination and anthropogenic factors may significantly contribute to the spread of resistance. Environmental stressors—such as sudden increases in water temperature and high turbidity caused by suspended organic matter—appeared to precede mortality peaks. The findings highlight the role of *Aeromonas* spp. as a key bacteria associated with mortality events in *S. trutta* and underscore the multifactorial nature of antibiotic resistance in aquaculture. No florfenicol-resistant isolates were detected in the farms where it is routinely used, indicating that florfenicol remains an effective antibiotic in aquaculture. However, the continuous and systematic monitoring of its use remains essential. The detection of bacteria not traditionally associated with fish pathology in samples from diseased animals suggests the need for further studies into their pathogenic potential. Overall, this descriptive study emphasizes the importance of preventive health strategies, prudent antibiotic use, and environmental monitoring to mitigate bacterial diseases and limit the spread of antimicrobial resistance in brown trout farming. These findings align with a One Health perspective, linking aquaculture practices, ecosystem integrity, and public health.

## Simple Summary

This study investigated bacterial isolates recovered from brown trout (*Salmo trutta*) during mortality events in five aquaculture farms in Spain: two of the farms had used antibiotics recently, while three had not applied any treatments in the six months prior to sampling. Tissue samples were collected from moribund fish showing clinical signs such as skin lesions and hemorrhages during outbreaks in 2022 and 2023. Bacterial identification was performed using real-time PCR, amplification and sequencing of the 16S rRNA gene and the ITS-1 intergenic spacer, and MALDI-TOF mass spectrometry. A total of 19 isolates were identified, with Gram-negative bacteria—particularly *Aeromonas* spp.—being the most frequently recovered. Some isolates belonged to genera not traditionally associated with fish disease, highlighting the need for further investigation into their role in fish health. Antibiotic-resistant strains were detected in four out of five farms, including those without recent antibiotic use, suggesting a potential contribution of environmental and anthropogenic factors to the spread of resistance. Florfenicol-resistant bacteria were not detected, indicating its continued effectiveness. Overall, the findings underscore the importance of preventive health measures, prudent antibiotic use, and environmental monitoring in brown trout aquaculture. The results support a One Health perspective by linking fish farming practices, environmental quality, and public health considerations.

## 1. Introduction

*Salmo trutta*, commonly known as the European brown trout, is a cold-water species that thrives in well-oxygenated waters below 15 °C, typically inhabiting cool rivers and streams. Its natural range includes Europe, North Africa, and Western Asia, but the species is now globally distributed due to human-mediated introductions. In Spain, three of the four main European mitochondrial lineages of brown trout are present: Atlantic, Mediterranean, and Duero, while the Adriatic lineage is absent. This species holds considerable ecological and economic value, particularly in recreational fishing and aquaculture. However, brown trout populations are vulnerable to various bacterial diseases that can cause significant mortality, affecting both wild stocks and aquaculture operations [1,2].

Accurate and rapid diagnostic techniques are essential for the effective management and prevention of bacterial diseases in brown trout populations. In Spain, the selection and validation of methods for detecting infectious diseases are guided by standards set by international and national organizations, as well as by specialized scientific literature [3,4].

The selection of appropriate diagnostic techniques depends on factors such as the age and species of the animals, observed clinical signs, available resources and tools, and the urgency of the diagnosis. Clinical examinations and necropsy are the initial steps in assessing the case and guiding subsequent diagnostic procedures. Within the diagnostic cascade, one of the fastest and most accurate methods for pathogen confirmation is real-time PCR (qPCR), which detects specific bacterial DNA or RNA from tissue, water, or cell culture samples. This technique is particularly valuable for identifying infections at early stages or when bacterial loads are low. Conventional PCR is similar to qPCR and also provides rapid results; however, it does not allow for quantification and requires confirmation through gel electrophoresis.

Techniques for bacterial confirmation and identification include isolation and culture, which allows for the assessment of bacterial morphology, virulence, and biochemical characteristics. Additionally, matrix-assisted laser desorption/ionization time-of-flight mass spectrometry (MALDI-TOF MS) enables rapid identification from pure culture colonies. 16S rRNA gene sequencing allows for fast comparison with databases, facilitating phylogenetic identification and providing information on the bacterial genus and, in some cases, species.

Among the most frequently detected bacterial pathogens in freshwater aquaculture, Gram-negative bacteria are particularly relevant. Most mortality outbreaks in the widely farmed rainbow trout (*Oncorhynchus mykiss*) are associated with *Aeromonas* spp., *Flavobacterium* spp., and *Pseudomonas* spp. [5]. Among these, *Aeromonas* infections are the most common. *Aeromonas salmonicida*, *A. sobria*, and *A. bestiarum* are frequently identified in such outbreaks, with *A. hydrophila* being the most significant pathogen due to the high mortality rates and economic losses it causes. *Aeromonas* species are ubiquitous and are also associated with foodborne and waterborne diseases that can affect mammalian species, including humans [6]. Other important pathogenic bacteria in freshwater fish include *Yersinia ruckeri*, *Lactococcus garvieae*, *Lactococcus petauri*, and *Carnobacterium maltaromaticum* [7].

Outbreaks of these and other infectious diseases are often triggered by environmental disturbances, such as the presence of pollutants or increases in water temperature that reduce dissolved oxygen levels, promote parasite infestations, or directly impair the physiological functions of the animals. Combined with poor management practices, such as overcrowding, these factors induce stress in fish and weaken their resistance to diseases [8].

Climate change is expected to create increasingly challenging conditions for *S. trutta* in Mediterranean river systems. Projections indicate rising temperatures, more frequent summer droughts, and changes in seasonal precipitation patterns [2]. Studies have shown that stress caused by acute or chronic changes in water temperature can alter the microbiome structure of salmonids, potentially compromising fish health [9].

Antibiotics are commonly used to treat bacterial infection outbreaks in fish within both commercial and restocking fish farms. These drugs are typically administered orally through specially formulated medicated feed to all fish in the same pond or batch, serving as a therapeutic or metaphylactic treatment to control and prevent the spread of infection. Among the most frequently used antibiotics are oxytetracycline, a broad-spectrum agent considered a first-line treatment but requiring high doses due to poor intestinal absorption in fish, and florfenicol, which has gained popularity in aquaculture because of its broad efficacy and lower risk of resistance development. Both antibiotics are particularly effective against diseases caused by Gram-negative bacteria. When administering these treatments, factors such as dosage, treatment duration, species, age, fish-health status, and water temperature must be taken into account. Flumequine is another antibiotic approved for use in aquaculture in Europe; however, as a quinolone, it is classified by the World Health Organization as a Highest Priority Critically Important Antimicrobial (HPCIA). Its use is reserved as a last resort option, as it belongs to a class of drugs that are essential for treating certain infections in hospitals, where a significant proportion of deaths is linked to fluoroquinolone resistance [10]. Antibiotic susceptibility testing (antibiograms) determines the resistance of isolated bacteria to various antibiotics and is essential for selecting appropriate treatments.

The skin, gills, and intestinal tract of aquatic animals are in direct contact with environmental bacteria. Therefore, detecting bacterial isolates carrying non-intrinsic antibiotic resistance genes in healthy animals or in those not exposed to antibiotics may indicate environmental antibiotic contamination or risk factors promoting the dissemination of resistant bacteria from nearby sources. Studies suggest that bacteria from the genera *Enterococcus* spp. and *Aeromonas* spp. could serve as indicators of antimicrobial resistance dissemination in aquatic environments: *Enterococcus* as a potential marker of fecal contamination and *Aeromonas* as a ubiquitous genus and one of the main groups of pathogens, particularly in freshwater fish [11].

Previous studies based on intestinal mucus samples from freshwater fish species indicate that anthropogenic activities contribute to the emergence and dissemination of antibiotic resistance genes. These genes are found not only in organisms from environments under direct selective pressure due to antibiotic use but also in ecosystems with minimal human intervention that are indirectly impacted by effluents from other anthropogenic sources [12]. This situation highlights the need to establish surveillance systems for the early detection of antibiotic resistance dissemination, and in this context, *S. trutta* could serve as an environmental bioindicator.

This is a descriptive and observational study aimed at identifying the predominant bacteria associated with increased mortality in *S. trutta* in Spanish restocking farms, including two farms with a recent history of antibiotic use and three with no antibiotic application in the six months prior to sampling. Additionally, we aimed to evaluate the antibiotic susceptibility profiles of the isolates, including both potential pathogens and commensal bacteria. This information is essential for developing preventive and therapeutic strategies to treat these diseases and to prevent the dissemination of antimicrobial resistance (AMR), using the most common and accessible techniques and tools available to the majority of veterinarians and diagnostic laboratories.

## 2. Materials and Methods

### 2.1. Farms Visited

**Figure 1:**
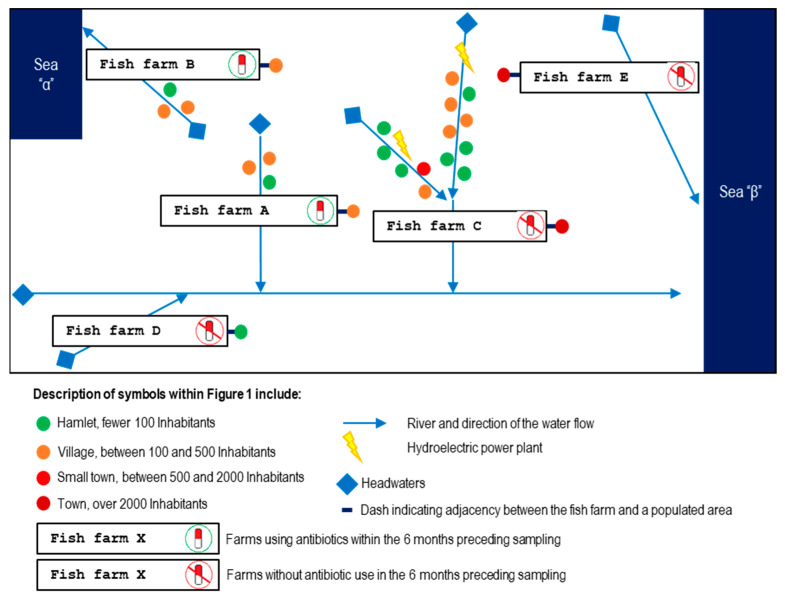
Diagram illustrating the relative locations of the five sampled *S. trutta* fish farms and the connecting river network (indicated by blue arrows). Farms A and B are those using florfenicol to treat bacterial infections, while farms C, D, and E did not use antibiotics. The rivers where these farms are located flow into two different seas, represented as “Sea α” and “Sea β,” thus highlighting the water flow.

Due to increased fish mortality, multiple visits were conducted during 2022 and 2023 to five *S. trutta* fish farms in Spain—designated as A, B, C, D, and E—which breed fish for river restocking. Each farm is located within a separate hydrographic basin. Although the rivers of three of these farms eventually converge into a larger main river downstream, there are no direct hydrological connections between the sampled farms. Furthermore, no other fish farms are located upstream of these sites (Figure 1).

Among them, the use of antibiotics in the six months prior to sampling was confirmed in Farms A and B through treatment records from the official farm register, showing that antibiotics had been administered orally via medicated feed to treat bacterial infections. In contrast, the other three farms had no recent history of antibiotic use according to their records.

Farm A is situated near a village with between 100 and 500 inhabitants, and its river is fed by tributaries that flow through two other villages and a hamlet before reaching the fish farm. The river then continues its course toward Sea α.

Farm B is also located near a village with between 100 and 500 inhabitants. Its upstream basin includes two villages and a hamlet, and its river eventually merges with a main river that flows into Sea β.

Farm C is located in a town with more than 2000 inhabitants and is situated in the most anthropogenically influenced basin in the study. Upstream, its river is regulated by two hydroelectric plants of different capacities (3.8 MW and 200 MW) and receives treated wastewater inputs from multiple human settlements, including seven hamlets (<100 inhabitants), five villages (100–500 inhabitants), and a small town (500–2000 inhabitants). The river originates in a high mountain area, merges with a main river, and its waters eventually reach Sea β.

Farm D is located near a hamlet with fewer than 100 inhabitants, and no significant human settlements exist in its upper basin, except for common grazing areas used for sheep and horses. This river later joins the main river, similar to those of Farms B and C, and ultimately flows into Sea β.

Finally, Farm E is situated near a town with more than 2000 inhabitants. Its upper basin is predominantly forested, with some scattered homes and no other significant urban areas. Its river originates in a high mountain region and flows directly into Sea β without any hydrological connection to the other fish farms in the study.

### 2.2. Sample Collection

**Figure 2:**
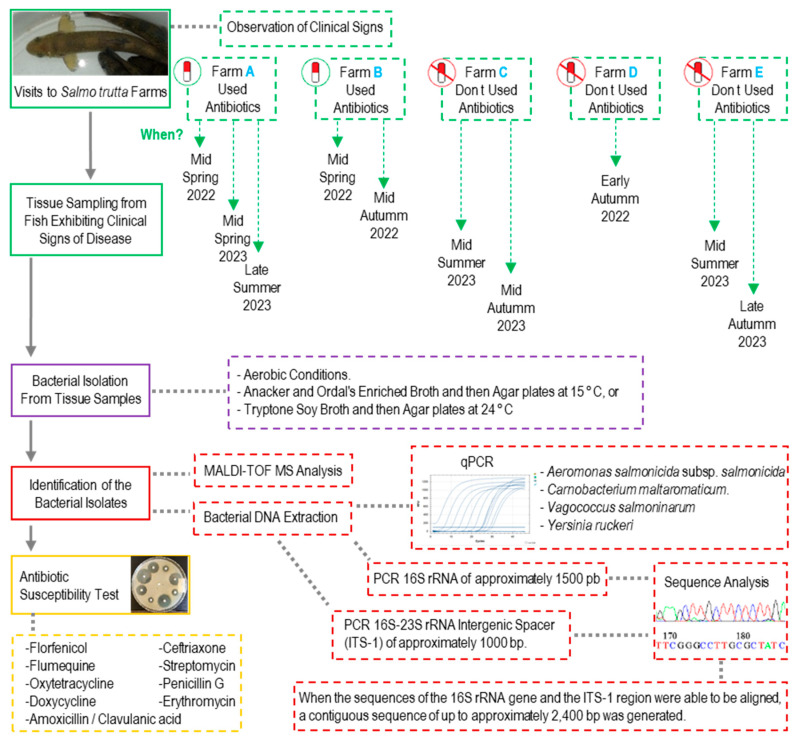
Schematic representation of the sampling, bacterial isolation, and molecular analysis workflow. This figure outlines the methodological approach used in this study. Fish exhibiting clinical signs of disease were selected for tissue sampling in five different farms (A–E) during multiple visits between 2022 and 2023. Farms A and B reported the use of florfenicol to treat bacterial infections, whereas Farms C, D, and E did not use antibiotics in the six months previous to the sampling. Bacterial isolates were obtained from the tissue samples under aerobic conditions using AOE broth at 15 °C or TSB at 24 °C and were subsequently identified through MALDI-TOF MS, qPCR targeting specific pathogens, and sequencing of the 16S rRNA gene and 16S-23S rRNA intergenic spacer (ITS-1). The bacterial isolates were also tested for antimicrobial susceptibility using disks of nine different antibiotics.

Figure 2 provides an overview of the methodological workflow used in this study. During each visit, moribund fish of any age or size (including yolk-sac fry, fingerlings, and adults) and those exhibiting clear clinical signs of disease (such as erratic swimming, ocular hemorrhages, fin hemorrhages, or skin lesions) were observed. All sampled fish were euthanized using tricaine methanesulfonate at a concentration of 180 mg/L (Tricaine Pharmaq 1000 mg/g, PHARMAQ AS, Overhalla, Norway) and buffered with phosphate buffer to pH 7. Cutaneous and gill mucus samples were collected to prepare smears and examine the presence of external parasites. Infected tissues—including skin, gills, liver, spleen, and/or the head kidney—were collected.

To obtain bacterial isolates from infected tissues, two strategies were used:

Tissue samples were plated directly onto Tryptone Soy Agar plates (TSA; Scharlab, Barcelona, Spain) at 24 °C or onto AOAE at 15 °C, and the plates were incubated for 12–72 h under aerobic conditions. Subsequently, a loopful of bacterial growth surrounding the tissue fragment was transferred to a tube containing Tryptone Soy Broth (TSB; Scharlab, Spain) at 24 °C or Anacker and Ordal’s Enriched Broth (AOE) at 15 °C. Cultures were incubated aerobically for 12–48 h with agitation at 100 rpm.

In yolk-sac or first-feeding fry, the yolk sac was removed (in yolk-sac fry), after which the fish were cut into small pieces and the tissues placed in centrifuge tubes containing 10 mL of TSB and incubated at 24 °C or AOE at 15 °C. Cultures were grown under aerobic conditions for 12 to 48 h with agitation at 100 rpm.

### 2.3. Bacterial Isolation from Infected Tissue Samples

After the initial incubation, serial 10-fold dilutions were performed on the bacterial cultures using sterile physiological saline (0.9% NaCl in distilled water). Subsequently, 100 μL from the 10^−7^, 10^−8^, 10^−9^, and 10^−10^ dilutions were plated onto Tryptone Soy Agar (TSA) or AOE agar plates. AOE agar plates were incubated at 15 °C and the TSA plates at 24 °C under aerobic conditions for 24 to 48–72 h. After incubation, three to five colonies per plate were selected, prioritizing the most abundant morphotypes, colonies whose appearance resembled that of known fish pathogens, and colonies of different sizes. Each colony was re-inoculated into TSB or AOE broth and incubated for 24–48 h at the same respective temperatures with agitation at 100 rpm. Drops from the resulting cultures were smeared on glass slides, air-dried, heat-fixed, stained using the Gram staining technique, and subsequently examined under the microscope.

Once the purity of each culture was confirmed, each bacterial isolate was assigned a unique identification code (e.g., St-Pi3-L-87) prior to species-level identification. Aliquots were stored at −80 °C in microcentrifuge tubes containing 15% glycerol until subsequent genomic DNA extraction and antibiotic susceptibility testing.

### 2.4. DNA Extraction and Identification of the Bacterial Isolates by qPCR

Total genomic DNA was extracted from 2 mL of each pure bacterial isolate culture using the QIAamp DNA Mini Kit™ (QIAGEN), following the manufacturer’s instructions [13]. qPCR assays were performed on bacterial isolates obtained from agar plates. Based on the microscopic morphology observed with Gram staining, Gram-positive isolates were tested for the presence of *Vagococcus salmoninarum* or *Carnobacterium maltaromaticum* [14,15], while Gram-negative isolates were tested for *A. salmonicida* subsp. *salmonicida* and for *Y. ruckeri* [16,17].

For qPCR, if an isolate tested positive, it was considered a definitive diagnosis, regardless of the other methods (Table 1).

### 2.5. Identification of the Bacterial Isolates by 16S rRNA and ITS-1 Sequencing

Conventional PCR was used to amplify the 16S rRNA gene using universal primers 27F and 1492R. Subsequently, the ITS-1 region, corresponding to the 16S-23S rRNA intergenic spacer, was amplified using primers R1391 and 473F [13]. PCR products were separated and visualized by agarose gel electrophoresis and purified from the gel using the NucleoSpin Gel and PCR Clean-Up Kit (Macherey-Nagel, Düren, Germany). The purified products were then sent to Macrogen, Spain (Madrid) for sequencing.

The forward and reverse sequences of the 16S rRNA and ITS-1 regions were analyzed using the BLAST tool on the NCBI website (https://blast.ncbi.nlm.nih.gov/Blast.cgi?PROGRAM=blastn&PAGE_TYPE=BlastSearch&LINK_LOC=blasthome, accessed on 29 June 2025), *Molecular Evolutionary Genetics Analysis* (MEGA) version 7.0.26 software, the reverse-complement.com online tool, and Microsoft Word for text processing. For nucleotide positions where the base identity could not be clearly determined, the appropriate letter representing degenerate bases was assigned according to IUPAC code recommendations. When possible, a single contiguous sequence comprising both the 16S rRNA and ITS-1 regions was assembled. The sequences obtained from the bacterial isolates were uploaded to the NCBI database.

For the 16S rRNA gene, sequences showing a ≥99% pairwise identity in BLAST were deemed reliable for species-level identification, whereas identities of ≥94.5% supported assignment at the genus level [18].

For the ITS-1 (16S–23S rRNA intergenic spacer), sequences with ≥99% identity were accepted for species-level identification and ≥94% for the genus level [19].

Sequences falling below these thresholds were considered non-identifiable.

**Table 1:** Sequences of PCR primers.

Target	Primer	Sequence 5′ to 3′	Amplicon/Reference
*Aeromonas salmonicida* subsp. *salmonicida*	aopP F	5′-CGGAACGTAATCTGAATTGTTCTTTTC-3′	340 pb [16]
	aopP R	5′-ATTGCTTATCGAGGCAGCCAAC-3′	
*Carnobacterium maltaromaticum*	16S F	5′-GAGGGTCATTGGAAACTGGA-3′	219 pb [14]
	16S R	5′-CGGAAACCCTCCAACACTTA-3′	
*Vagococcus salmoninarum*	sal F	5′-GACGCTTTCGGGTGTCACTA-3′	543 pb [15]
	sal R	5′-CAGACCAGAGAGTCGCCTTC-3′	
*Yersinia ruckeri*	glnA F	5′-TCCAGCACCAAATACGAAGG-3′	113 pb [17]
	glnA R	5′-ACATGGCAGAACGCAGATC-3′	
	glnA P	HEX-5′-AAGGCGGTTACTTCCCGGTTCC-3′-BHQ1	
16S rRNA gene	27F	5′-AGAGTTTGATCCTGGCTCAG-3′	1465 pb [20]
	1492R	5′-ACGGCTACCTTGTTACGACTT-3′	
16S rRNA-23S rRNA intergenic spacer	R1391	5′-TTGTACACACCGCCCGTC-3′	1000 pb [13]
	473F	5′-TTGTACACACCGCCCGTC-3′	

### 2.6. Identification of the Bacterial Isolates by MALDI-TOF MS

The bacterial isolates were sent to a commercial veterinary diagnostic laboratory for identification using MALDI-TOF MS. The analysis was performed using a Bruker MALDI Biotyper system (Bruker Daltonics GmbH, Bremen, Germany), following standard protocols. At the laboratory, pure bacterial colonies were analyzed using MALDI-TOF MS in accordance with the manufacturer’s instructions and the laboratory’s standard operating procedures. The generated mass spectra were compared against the reference database provided by the manufacturer to determine the species-level identification.

Identification scores obtained from the MALDI-TOF MS analysis were interpreted as follows: isolates with a score of ≥2.00 were considered reliably identified at the species level; scores between 1.70 and 1.99 were considered reliable for the genus level; and scores below 1.70 were considered unreliable [21].

It is important to note that both MALDI-TOF MS and 16S rRNA sequencing have limitations in differentiating certain bacterial species. For MALDI-TOF MS, limitations may arise due to incomplete protein profile databases, the absence of specific entries leading to genus-level identification without precise species assignment, or an increased risk of misidentification when expanding the range of species analyzed. In the case of 16S sequencing, its discriminatory power is limited for species within the same genus due to the high similarity of their sequences [22].

### 2.7. Phylogenetic Analysis

To assess the phylogenetic relationships of the isolates, the 16S rRNA sequences were compared with reference sequences from previously described type strains. The complete list of reference sequences used, along with their GenBank accession numbers, is provided in Table 2.

A phylogenetic tree was constructed using the Maximum Likelihood (ML) method in MEGA7 [23]. Since some isolates were sequenced only for the 16S rRNA gene, the phylogenetic reconstruction was based exclusively on this genetic region, excluding longer fragments that included the 16S-23S intergenic regions. This approach ensured a homogeneous comparison among all samples, minimized biases due to fragment length differences, and facilitated the inclusion of reference sequences from public databases, most of which are based on the 16S rRNA gene.

The phylogenetic analysis was conducted under the General Time Reversible (GTR) model [24], selected as the best-fit model based on likelihood criteria. A Gamma distribution with four rate categories (+G, parameter = 0.3828) was used to model the rate variation among sites, and a proportion of sites was considered invariable ([+I], 32.53%). The initial tree topology for the heuristic search was obtained using the Neighbor-Joining (NJ) and BioNJ algorithms, based on a distance matrix estimated with the Maximum Composite Likelihood (MCL) approach, and the topology with the highest log likelihood was selected.

### 2.8. Antibiotic Susceptibility Test

Fresh pure cultures of bacteria isolated from fish tissues, including both potential pathogenic and commensal bacteria that could serve as indicators of antimicrobial resistance, were used to perform antibiotic susceptibility testing using disks impregnated with nine different antibiotics (Oxoid, Hampshire, UK): amoxicillin/clavulanic acid (30 µg), doxycycline (30 µg), erythromycin (15 µg), florfenicol (30 µg), ceftriaxone (30 µg), flumequine (30 µg), penicillin G (10 units), streptomycin (10 µg), and oxytetracycline (30 µg).

Fresh bacterial cultures were centrifuged at 2500× *g* for 10 min. The supernatant was discarded, and the bacteria was resuspended in a 0.9% NaCl solution to a turbidity equivalent to 0.5 McFarland, corresponding to 1.5 × 10^8^ CFU mL^−1^. Bacteria were seeded in triplicate onto Mueller Hinton Agar (Bio-Rad, France) plates (150 mm × 25 mm) using a sterile swab. The antibiotic disks were placed on the surface of the agar, and the plates were incubated aerobically at 24 °C for 18 to 48 h, depending on the bacterial growth rate. After incubation, the zones of inhibition around each antibiotic disk were measured in millimeters (mm), recording the diameter of the clear zone where bacterial growth was inhibited.

The measured inhibition zones and clinical breakpoints were interpreted according to the Clinical and Laboratory Standards Institute (CLSI) guidelines: M100 *Performance Standards for Antimicrobial Susceptibility Testing* [25], M45 *Methods for Antimicrobial Dilution and Disk Susceptibility Testing of Infrequently Isolated or Fastidious Bacteria* [26], and VET04 *Performance Standards for Antimicrobial Susceptibility Testing of Bacteria Isolated from Aquatic Animals* [27]. These guidelines classify the bacterial strains as susceptible (S), intermediate (I), or resistant (R) based on the diameter of the inhibition zones.

For cases where no CLSI guidelines were available, if no inhibition zone was observed, the bacterial strain was recorded as resistant (R) to the antibiotic, indicating clear in vitro resistance. When guidelines were not available and the inhibition zone diameter was equal to or greater than 25 mm, the strain was recorded as susceptible (S) based on general trends and interpretations from available guidelines. For inhibition zones smaller than 25 mm without specific references, the results were recorded as not determined (ND), indicating that no definitive conclusion could be drawn regarding susceptibility.

For certain species, we also used epidemiological cutoff values (ECV) from the VET04 manual. ECVs help distinguish between wild-type populations and those with acquired or mutational resistance. For species where ECVs were available, the smallest inhibition zone diameter defining the wild-type population was used to differentiate wild-type strains from those with potential resistance mechanisms.

## 3. Results

### 3.1. Fish Farm A: Observations and Bacterial Diagnosis

#### 3.1.1. First Visit, Mid-Spring 2022

Two weeks prior to the onset of clinical signs in the fish, the water temperature increased by approximately 4 °C above the usual levels. According to treatment records in the official farm register, Farm A had administered florfenicol orally via medicated feed in the months prior to sampling, as part of the therapeutic management of high mortality episodes. However, no antibiotic treatments were ongoing at the time of sampling.

**Figure 3:**
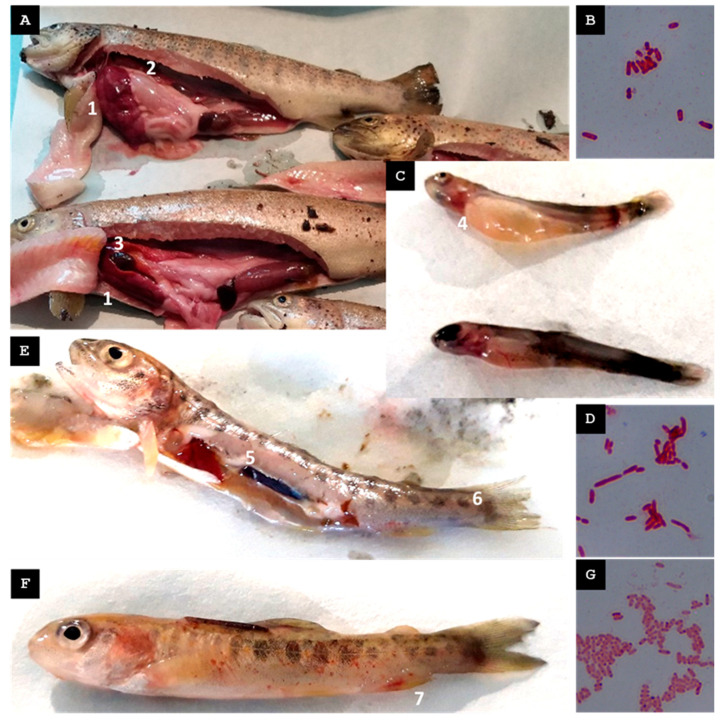
Farm A. (**A**) Female broodstock captured during the first visit (mid-spring 2022) exhibiting hepatomegaly (1), ascitic fluid in the peritoneal cavity with pathological gastric dilation (2), and gallbladder distension (3). (**B**) Gram-stained isolate St-Pi3-L-87. (**C**) Sac fry collected during the second visit (mid-spring 2023) displaying caudal melanosis and redness with hemorrhagic lesions around the head and gill region (4). (**D**) Gram-stained isolate St-Pi3-VL-99. (**E**) Fry from the third visit (late summer 2023) showing an enlarged posterior kidney (5) and erosions on the caudal fin (6). (**F**) Fry from the third visit exhibiting petechial hemorrhages along the lateral line and the ventral area adjacent to the anal fin (7). (**G**) Gram-stained isolate St-Pi3-OP-104.

Five female broodstock exhibiting erratic swimming behavior were captured, and the following pathological findings were observed: ascitic fluid in the peritoneal cavity, hepatomegaly, hepatic hemorrhages, stomach dilation (without food), and gallbladder distension (Figure 3A).

Among the bacteria isolated from pooled hepatic tissue samples of five specimens cultured in TSB, only one could be properly identified, a Gram-negative bacterium, designated St-Pi3-L-87 (Figure 3B).

The MALDI-TOF mass spectrometry score was <1.70, indicating that the isolate could not be reliably identified by this method. The 16S rRNA gene sequence yielded a fragment of 1405 nucleotides and showed 97.30% identity to *Plesiomonas shigelloides*. The ITS-1 sequence yielded a fragment of 766 nucleotides and showed 92.48% identity to *P. shigelloides*. Both fragments were assembled into a contiguous 2102-nt sequence. BLAST analysis of this sequence (NCBI) revealed the highest similarity to *P. shigelloides*, with 95.49% identity and 100% query coverage. Given the low identity values and the absence of a reliable MALDI-TOF MS match, the isolate St-Pi3-L-87 was provisionally classified as *Plesiomonas* sp., and further studies are required for confirmation.

#### 3.1.2. Second Visit, Mid-Spring 2023

One week before clinical signs emerged, a slight rise in water temperature was re-corded, followed by increased mortality in sac fries in the days preceding the site visit. During the visit, ten yolk sac fries were collected. Fish from both groups exhibited caudal melanosis and hemorrhagic lesions around the head and gill regions (Figure 3C).

From the tissue of the sac fry, which was cut into small fragments and placed in TSB for bacterial isolation, only one of the predominant colonies could be properly identified. Its morphology resembled that typically observed in *Aeromonas* species, and it was identified as a Gram-negative bacterium, designated St-Pi3-VL-99 (Figure 3D).

An MALDI-TOF MS analysis identified this isolate as *A. sobria* with a score of 1.99. Amplification of the 16S rRNA gene from the total genomic DNA of the isolate yielded a 1423-nucleotide fragment, which, upon BLAST analysis, showed 100% identity to *A. salmonicida*. Only a small sequence of the ITS-1 region (593-nt) was obtained and showed 99.83% identity to *A. salmonicida*. The MALDI-TOF score only supported genus-level confidence, so the identification of the isolate St-Pi3-VL-99 was conservatively restricted to the genus as an *Aeromonas* sp.

#### 3.1.3. Third Visit, Late Summer 2023

In the days prior to the visit, an increase in water temperature supplying the fish farm was recorded, followed by a rise in mortality in fry. Captured fry, *n* = 20, exhibited erratic swimming. Fish showed gill redness, petechial hemorrhages along the lateral line, liver hemorrhages, and inflammation of the caudal kidney portion. (Figure 3E,F).

From the colonies obtained from pooled organ samples (spleen, kidney, and liver) grown in TSB, only one Gram-negative bacterium designated St-Pi3-OP-104 (Figure 3G) was reliably identified. An MALDI-TOF MS analysis identified this isolate as *Hafnia alvei* with a score of 2.03, indicating reliable species-level identification. Amplification and sequencing of the 16S rRNA gene yielded a 1392-nt fragment that showed 100% identity query coverage with *Hafnia alvei* in an NCBI BLAST search. In addition, a 525-nt fragment of the ITS-1 region was successfully sequenced and exhibited 99.43% identity to *H. alvei.* Based on these results, isolate St-Pi3-OP-104 was considered reliably identified as *H. alvei*.

### 3.2. Fish Farm B: Observations and Bacterial Diagnosis

#### 3.2.1. First Visit, Mid-Spring 2022

Two weeks before the mortality spike, an increase in the turbidity of the river water supplying the farm was recorded. According to the treatment records in the official farm register, Farm B had administered florfenicol orally via medicated feed occasionally in the months preceding the visit, specifically in response to episodes of elevated mortality. However, no antibiotic treatments were being applied at the time of sampling.

**Figure 4:**
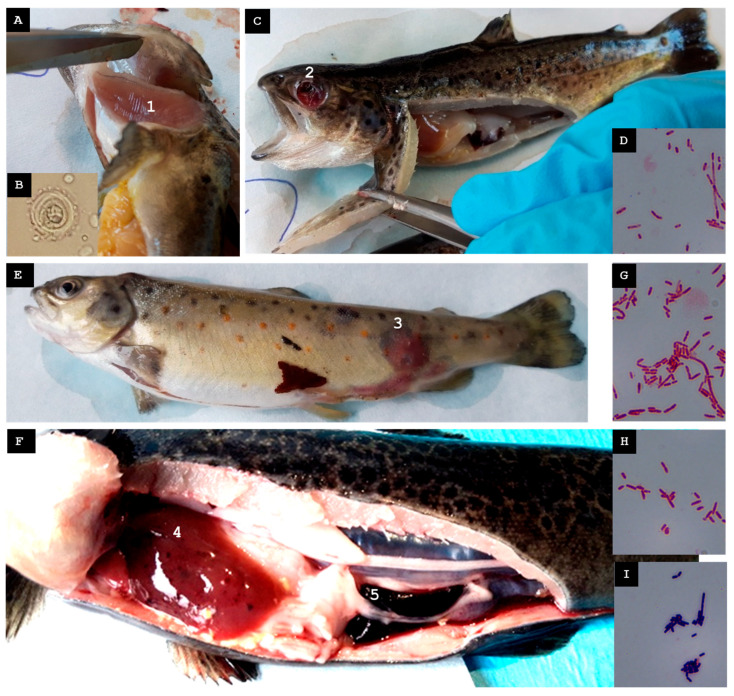
Farm B. (**A**) Juvenile from the first visit (mid-spring 2022) exhibiting gill pallor (1). (**B**) *Trichodina* sp. observed on the gills of the specimen shown in panel A. (**C**) Juvenile from the first visit showing ocular rupture as a result of exophthalmos (2). (**D**) Gram-stained isolate St-Pi5-Sp-107. (**E**) Juvenile from the second visit (mid-autumn 2022), batch Pi5.L1, presenting a furuncle (3) and subcutaneous hemorrhages along the ventral region. (**F**) Adult fish from the second visit, batch Pi5.L2, displaying melanosis, hepatomegaly with widespread petechial hemorrhages (4), and splenomegaly (5). (**G**) Gram-stained isolate St-Pi5-Sk-98. (**H**) Gram-stained isolate St-Pi5-Sk-89. (**I**) Gram-stained isolate St-Pi5-L-92.

Ten moribund or erratically swimming juveniles were collected (Figure 4A). A mild *Trichodina* sp. infestation was observed on the gills (Figure 4B). Some fish exhibited ocular hemorrhages or the loss of one or both eyeballs, gill pallor, hemorrhagic skin lesions, hepatomegaly, and gastric dilation with mucous content (Figure 4C).

A Gram-negative bacterium, designated St-Pi5-Sp-107, isolated from pooled spleen tissue, was the only isolate that could be reliably identified (Figure 4D). MALDI-TOF MS assigned it to *Aeromonas encheleia* with a score of 1.99, indicating reliable genus-level identification. Amplification and sequencing of the 16S rRNA gene produced a 1408-nt fragment sharing 99.93% identity with *A. encheleia*, whereas sequencing of the ITS-1 region produced a 150-nt fragment showing 100% identity with the same species. Alignment of the ITS-1 fragment with the 16S rRNA sequence generated a continuous 1538-nt contig that exhibited 100% query coverage with *A. encheleia* in a BLAST search. Taken together, these results confirmed isolate St-Pi5-Sp-107 as *A. encheleia*.

#### 3.2.2. Second Visit, Mid-Autumn 2022

In the week preceding the visit, mortality at the farm increased. Ten juveniles from batch Pi5.L1 and one recently deceased adult (>2 years old) from batch Pi5.L2 were collected. Juveniles exhibited furuncles, petechiae in the liver and intestines, and splenomegaly (Figure 4E). The adult displayed melanosis and petechial hemorrhages in the liver and intestines (Figure 4F).

From the skin furuncles of juveniles of batch Pi5.L1, two Gram-negative colonies, designated St-Pi5-Sk-98 (Figure 4G) and St-Pi5-Sk-89 (Figure 4H), were reliably identified. From the hepatic tissue of the adult fish from batch Pi5.L2, only one Gram-positive colony, designated St-Pi5-L-92, could be identified (Figure 4I).

Isolate St-Pi5-Sk-89 tested qPCR positive for *A. salmonicida* subsp. *salmonicida.* In MALDI-TOF MS analysis, it scored 1.72 as *A. bestiarum*, allowing for reliable identification at the genus level (*Aeromonas* sp). The 16S rRNA sequence (1420 nt) exhibited 100% identity with *A. salmonicida*, while the ITS-1 sequence (1058 nt) showed 97.36% identity with *A. bestiarum*. Alignment of the two fragments produced a 2437-nt contig. Isolate St-Pi5-Sk-89 was identified as *A. salmonicida* subsp. *salmonicida*.

Isolate St-Pi5-L-92 had a qPCR positive result for *C. maltaromaticum*. In MALDI-TOF MS analysis, it scored 2.41 as *C. maltaromaticum*. The BLAST analysis of the 16S rRNA sequence (1413 nt) revealed 100% identity with *C. maltaromaticum*; the ITS-1 sequence (1009 nt) also shared 99.41% identity with *C. maltaromaticum*. The sequences were combined into a 2384-nt contig. Isolate St-Pi5-L-92 was identified as *C. maltaromaticum*.

Isolate St-Pi5-Sk-98 scored 2.22 as *Pseudomonas fulva* in MALDI-TOF MS analysis. The BLAST analysis of the 16S rRNA sequence (1412 nt) showed 100% identity with *P. fulva*, whereas the ITS-1 sequence (789 nt) displayed 99.62% identity with the same species. The two sequences were assembled into a single 2162-nt contig. Isolate St-Pi5-Sk-98 was identified as *P. fulva*.

### 3.3. Fish Farm C: Observations and Bacterial Diagnosis

#### 3.3.1. First Visit, Mid-Summer 2023

**Figure 5:**
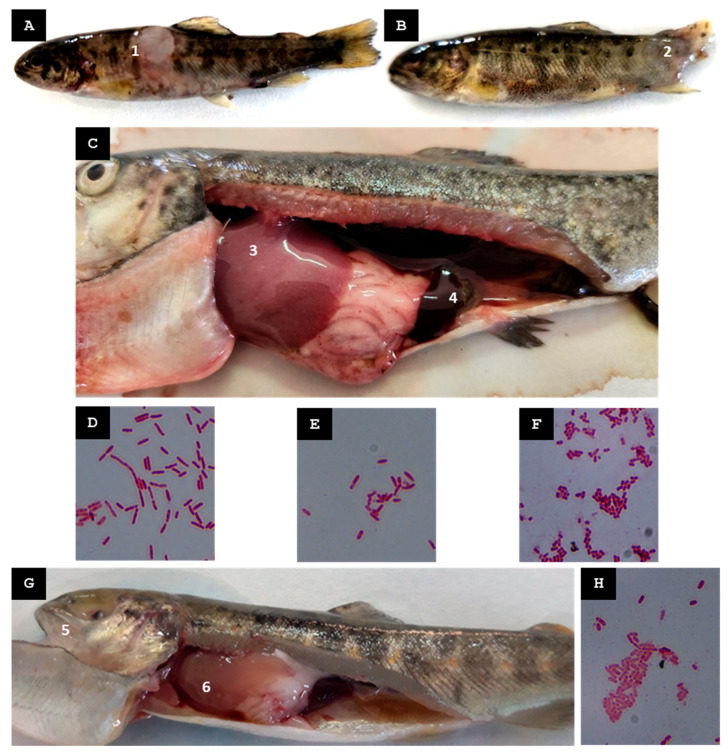
Farm C. (**A**) Fry from the first visit (mid-summer 2023), batch Pi6.L1, displaying deep cutaneous wounds with loss of muscle tissue (1). (**B**) Fry from the first visit, batch Pi6.L1, showing complete erosion and disappearance of the caudal fin (2). (**C**) Juvenile from the first visit, batch Pi6.L2, exhibiting hepatomegaly (3) together with splenomegaly (4). (**D**) Gram-stained isolate St-Pi6-OP-110. (**E**) Gram-stained isolate St-Pi6-OP-111. (**F**) Gram-stained isolate St-Pi6-OP-113. (**G**) Juvenile from the second visit (mid-autumn 2023), presenting loss of the ocular globe (5) along with hepatomegaly and liver discoloration (6). (**H**) Gram-stained isolate St-Pi6-OP-105.

Eight moribund fries measuring 4–6 cm in length were collected from batch Pi6.L1, along with eighteen juveniles measuring between 9 and 14 cm from batch Pi6.L2. Fry in batch Pi6.L1 exhibited deep cutaneous ulcers with the loss of muscle tissue and, in some cases, complete erosion and disappearance of the caudal fin (Figure 5A,B). In batch Pi6.L2, internally, hepatomegaly and splenomegaly were observed in some specimens (Figure 5C).

Two Gram-negative colonies were successfully identified from pooled liver tissue and cutaneous lesions of fish in batch Pi6.L1 that had been inoculated in TSB and AOE; these were designated St-Pi6-OP-110 (Figure 5D) and St-Pi6-OP-111 (Figure 5E). From pooled organ samples (liver, kidney, spleen, and ocular lesions) of fish in batch Pi6.L2, only a single Gram-negative colony could be reliably identified, designated St-Pi6-OP-113 (Figure 5F).

For isolate St-Pi6-OP-110, the MALDI-TOF MS analysis scored 1.76 as *A. bestiarum*, supporting reliable genus-level identification (*Aeromonas* sp.). A 1376-nt fragment of the 16S rRNA gene was successfully amplified; BLAST analysis showed 100% identity with *A. salmonicida*. The ITS-1 region yielded a 616-nt fragment that shared 99.83% identity with *A. bestiarum*. Isolate St-Pi6-OP-110 was identified at the genus level as *Aeromonas* sp.

For isolate St-Pi6-OP-111, the MALDI-TOF MS analysis scored a 2.01 as *A. hydrophila*. The 16S rRNA sequence (1391 nt) displayed 100% identity with *A. hydrophila*, while a 152-nt ITS-1 fragment showed 99.83% identity with the same species. Alignment of the two fragments generated a 1528-nt contig. Isolate St-Pi6-OP-111 was identified at the species level as *A. hydrophila*.

For isolate St-Pi6-OP-113, the MALDI-TOF MS analysis scored a 2.06 as *Kluyvera intermedia*. The 16S rRNA sequence (1408 nt) showed 100% identity with *Kluyvera intermedia*, and the 886-nt ITS-1 sequence exhibited 98.31% identity with that species. Assembly of these fragments produced a 2274-nt contig. Isolate St-Pi6-OP-113 was confirmed as *K. intermedia*.

#### 3.3.2. Second Visit, Mid-Autumn 2023

Several days before lesions became visible, heavy rainfall increased the turbidity of the inflowing water. Thirteen juveniles (8.5–10 cm) were sampled; these fish exhibited the rupture of one or both eyeballs, hepatomegaly, and liver discoloration (Figure 5G).

From a pooled organ sample (liver, kidney, spleen, and muscle) only one Gram-negative colony, designated St-Pi6-OP-105, was reliably identified (Figure 5H).

MALDI-TOF MS assigned this isolate to *Kluyvera* spp. with a score of 1.74, indicating genus-level reliability. Sequencing of the 16S rRNA gene (1397 nt) revealed 99.93% identity to *Kluyvera intermedia* in the BLAST analysis, and the ITS-1 fragment (542 nt) showed 99.08% identity with the same species. Taken together, these results confirm isolate St-Pi6-OP-105 as *Kluyvera intermedia*.

### 3.4. Fish Farm D: Observations and Bacterial Diagnosis

#### Visit in Early Autumn 2022

**Figure 6:**
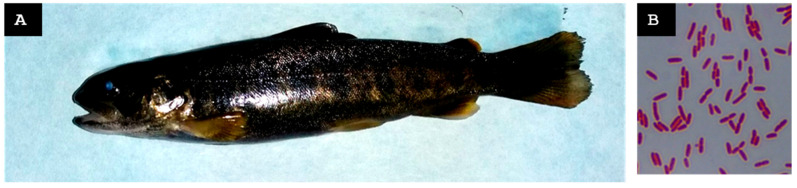
Farm D. (**A**) *S. trutta* specimen, collected during the early autumn 2022 visit, displaying melanosis. (**B**) Gram-stained isolate St-Pi2-Sp-101.

Following reports of elevated mortality during the previous week, ten juveniles displaying erratic swimming and generalized melanosis were collected (Figure 6A). No internal lesions were observed in these fish.

Pooled liver and spleen tissues were cultured in TSB. Only one Gram-negative isolate, designated St-Pi2-Sp-101 (Figure 6B), could be reliably identified.

MALDI-TOF MS assigned this isolate to *Aeromonas eucrenophila* with a score of 2.05. Sequencing of the 16S rRNA gene produced a 1419-nt fragment that showed 99.86% identity to *A. salmonicida* in the BLAST analysis, whereas the ITS-1 fragment obtained (963 nt) shared 98.03% identity with *A. bestiarum*. Taken together, these results support the classification of isolate St-Pi2-Sp-101 as *Aeromonas* sp.

### 3.5. Fish Farm E: Observations and Bacterial Diagnosis

#### 3.5.1. First Visit, Mid-Summer 2023

**Figure 7:**
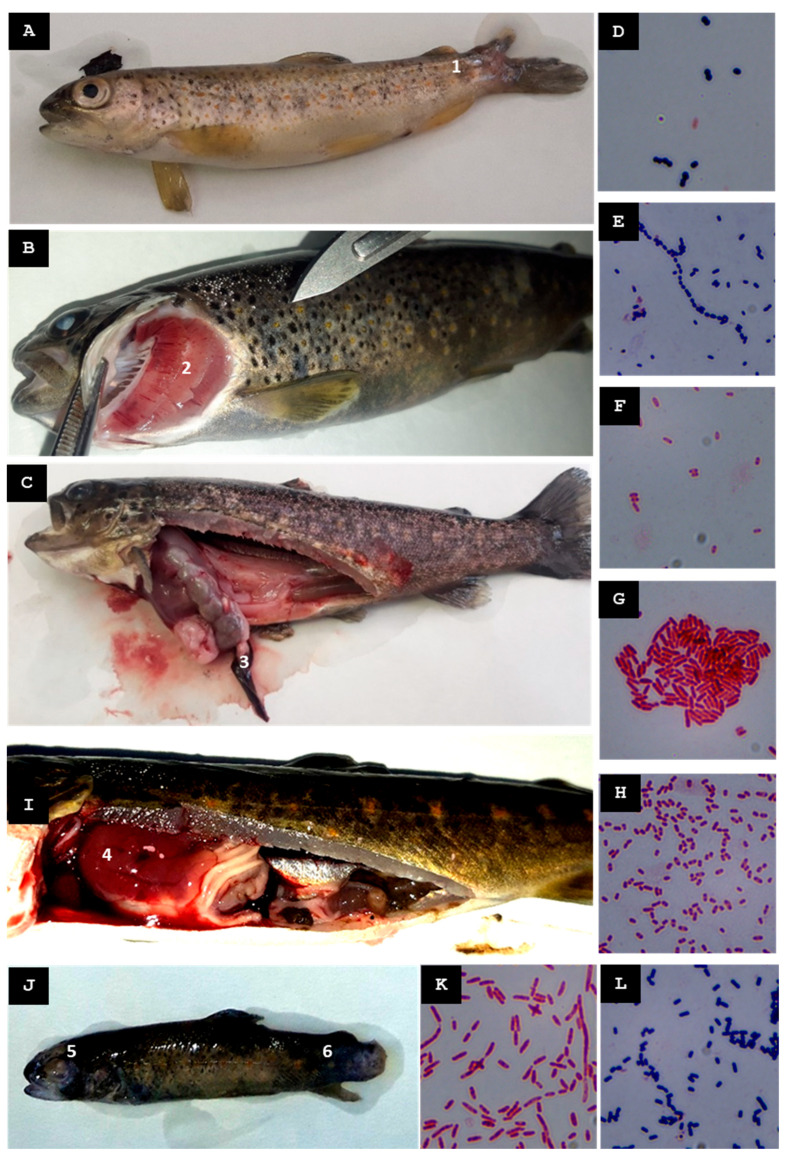
Clinical signs observed at Farm E. (**A**) Juvenile from the first visit (mid-summer 2023), from batch Pi7.L1, presenting fin-tissue loss (1). (**B**) Juvenile from the first visit, batch Pit.L2, presenting gill petechiae (2). (**C**) Juvenile from the first visit, batch Pi7.L3, exhibiting splenomegaly (3). (**D**) Gram-stained isolate St-Pi7-OP-96. (**E**) Gram-stained isolate St-Pi7-OP-114. (**F**) Gram-stained isolate St-Pi7-OP-115. (**G**) Gram-stained isolate St-Pi7-OP-97. (**H**) Gram-stained isolate St-Pi7-OP-116. (**I**) Juvenile from the second visit (late autumn 2023) exhibiting hepatomegaly with visible hepatic hemorrhages (4). (**J**) Juvenile from the second visit, showing ruptured eyeballs (5) and erosion of the caudal fin (6). (**K**) Gram-stained isolate St-Pi7-OP-119. (**L**) Gram-stained isolate St-Pi7-OP-118.

Nine juveniles measuring 9–10 cm in length were collected from batch Pi7.L1, along with four recently deceased juveniles (12–16 cm) from batch Pi7.L2 and four moribund juveniles (17–18 cm) from batch Pi7.L3. Juveniles in batch Pi7.L1 exhibited cutaneous lesions with the loss of muscle and/or fin tissue (Figure 7A), but no internal organ lesions were detected. In batch Pi7.L2, dorsal-fin hemorrhages and gill petechiae were observed (Figure 7B); internal examination revealed a distended gallbladder and abundant mucous gastric contents. In batch Pi7.L3, several specimens displayed fin hemorrhages, and splenomegaly was recorded in two fish (Figure 7C).

From pooled tissue samples of batch Pi7.L1 (liver, spleen, kidney, and skin lesion), three isolates were reliably identified: two Gram-positive cocci, St-Pi7-OP-96 (Figure 7D) and St-Pi7-OP-114 (Figure 7E), and one Gram-negative bacillus, St-Pi7-OP-115 (Figure 7F). From pooled organ samples of skin lesions, the kidney, liver, and spleen from batch Pi7.L2, a single Gram-negative bacillus, St-Pi7-OP-97 (Figure 7G), was accurately identified. Finally, from the pooled liver, kidney and spleen samples of batch Pi7.L3, only one Gram-negative bacterium, St-Pi7-OP-116 (Figure 7H), was reliably identified.

For isolate St-Pi7-OP-96, the MALDI-TOF MS analysis scored a 2.12 as *Enterococcus faecium*. The 16S rRNA sequence (1432-nt) displayed a 100% identity to *E. faecium*. The ITS-1 sequence (965-nt) showed 100% identity to *E. faecium*. Alignment of the two fragments generated a 2337-nt contig. Isolate St-Pi7-OP-96 was identified as *E. faecium*.

For isolate St-Pi7-OP-114, the MALDI-TOF MS analysis scored 1.71 as *Lactococcus raffinolactis*: the 16S rRNA sequence (1045-nt) showed 100% identity to *L. raffinolactis*. The ITS-1 sequence (974-nt) showed 99.18% identity. Alignment of the sequences generated a 2312-nt contig. Isolate St-Pi7-OP-114 was identified as *L. raffinolactis*.

For isolate St-Pi7-OP-115, the MALDI-TOF MS analysis scored a 2.05 as *Hafnia alvei*. The 16S rRNA sequence (1404-nt) displayed 100% identity to *H. alvei*. The ITS-1 sequence (992-nt) showed 98.59% identity. Both sequences were assembled into a 2375-nt contig. Isolate St-Pi7-OP-115 was identified as *H. alvei*.

For isolate St-Pi7-OP-97, the MALDI-TOF MS analysis scored a 1.92 as A. bestiarum. The 16S rRNA sequence (1383-nt) showed 100% identity to *Aeromonas sobria*, and the ITS-1 (996-nt) showed 96.79% identity to *A. salmonicida.* Alignment of the sequences generated a 2378-nt contig. Isolate St-Pi7-OP-97 was identified as *Aeromonas* sp.

For isolate St-Pi7-OP-116, the MALDI-TOF MS analysis scored a 2.15 as *H. alvei*. The 16S rRNA sequence (1401-nt) displayed a 100% identity to *H. alvei*, and the ITS-1 (965-nt) showed a 98.55% identity. Both sequences assembled into a 2358-nt contig. Isolate St-Pi7-OP-116 was identified as *H. alvei*.

#### 3.5.2. Second Visit, Late Autumn 2023

During the visit, ten juvenile fish were collected; several showed ruptured eyeballs, caudal fin erosion, and hepatomegaly (Figure 7I,J).

From pooled liver and kidney samples, two isolates were reliably recovered: a Gram-positive bacterium designated St-Pi7-OP-118 (Figure 7L) and a Gram-negative bacterium designated St-Pi7-OP-119 (Figure 7K).

Isolate St-Pi7-OP-118 tested positive by qPCR for *C maltaromaticum*. The 16S rRNA (1398 nt) showed 99.93% identity to *C. maltaromaticum*, and the ITS-1 (980 nt) showed 99.18% identity to the same species. Both fragments assembled into a 2343-nt contig. These data confirmed this isolate as *C. maltaromaticum*.

Isolate St-Pi7-OP-119 was assigned by MALDI-TOF MS to *Aeromonas bestiarum* (score of 1.83), i.e., reliable genus-level identification (*Aeromonas* sp.). The 16S rRNA (1371 nt) showed 100% identity to *A. salmonicida*, and the ITS-1 (1054 nt) showed 96.58% identity to *A. bestiarum*. The two fragments assembled into a 2408-nt contig. This isolate was identified as *Aeromonas* sp.

All identification results are integrated in Table 3.

### 3.6. Phylogenetic Analysis

**Figure 8:**
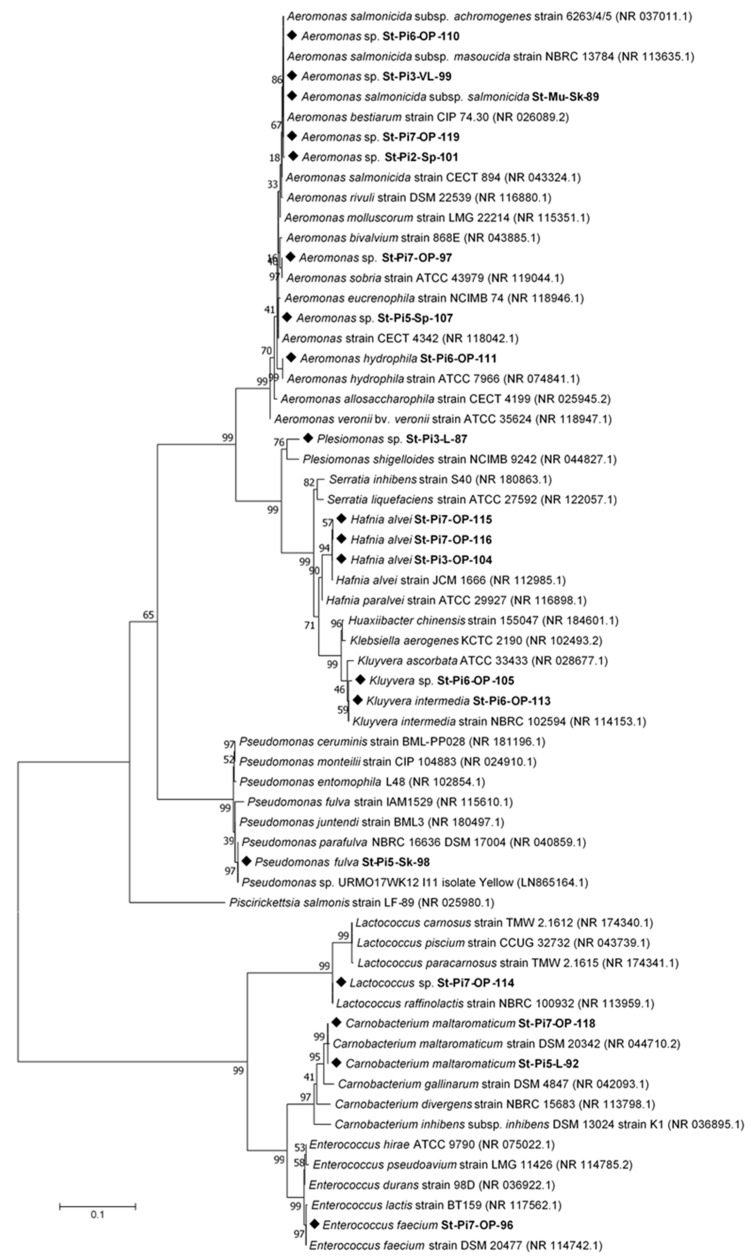
Maximum likelihood phylogenetic tree based on 16S rRNA gene sequences showing the relationships of bacterial isolates obtained in this study (codes in bold) and reference strains retrieved from GenBank (accession numbers beginning with NR; see Table 2). Numbers at the nodes represent bootstrap support values (%). The analysis was performed in MEGA7 using the GTR+G+I model. Sequences obtained in this study are shown in bold and marked with a solid diamond (◆).

The maximum likelihood phylogenetic tree with the highest log likelihood (−7353.37) is presented in Figure 8. The analysis comprised 62 nucleotide sequences, yielding a final alignment of 1325 positions after excluding sites with <95% coverage (i.e., positions containing >5% gaps, missing data, or ambiguous bases were removed).

### 3.7. Antibiotic Susceptibility Test

Interpretation of susceptibility results followed CLSI documents M100, M45, and VET04 [25,26,27]; where no guidance was available, the criteria described in Section 2.8 were applied. Results are shown in Table 4.

At least one isolate resistant to one or more of the tested antibiotics was detected in farms A, B, C, and E. In Farm D, only a single isolate was recovered, and it displayed no resistance beyond that considered intrinsic to the species.

Regarding antibiotics commonly used in aquaculture, florfenicol resistance was found in one isolate from Farm E (*Aeromonas* sp. St-Pi7-OP-97); oxytetracycline resistance in one isolate from Farm C (*A. hydrophila* St-Pi6-OP-111) and in one isolate from Farm E of *S. trutta* (*E. faecium* St-Pi7-OP-96); and flumequine resistance in one isolate from Farm E of *S. trutta* (*L. raffinolactis* St-Pi7-OP-114).

Using the antibiotic panel applied here, no multidrug-resistant (MDR) bacteria were detected among the *S. trutta* isolates, i.e., none met the criterion of resistance to at least one agent in three different antimicrobial classes [28].

Table 5 compiles and summarizes, by farm, the approximate human population living upstream and in the immediate vicinity of the fish farm, the season during which the visit and sampling took place, the main clinical signs observed in diseased fish, the bacterial isolates that were reliably identified, and the antibiotics to which they exhibited non-intrinsic resistance.

## 4. Discussion

This study characterizes bacterial isolates associated with mortality events in *S. trutta* (brown trout) across five Spanish fish farms and examines their potential antibiotic resistance profiles. Gram-negative bacteria were the predominant group linked to mortality in *S. trutta*, consistent with the general observation that Gram-negative pathogens are most frequently implicated in mass-mortality episodes in freshwater aquaculture systems [5].

Across farms and sampling visits, the most common clinical signs were hepatic pathology, ocular lesions, hemorrhages, fin erosion, and cutaneous lesions. The primary putative bacterial agents were *A. salmonicida*, *A. hydrophila*, *A. encheleia*, and other *Aeromonas* spp. In this study, *Aeromonas* spp. accounted for 42% of all isolates, reinforcing their well-documented role as opportunistic fish pathogens and their potential impact on food and water-borne diseases [5,6]. *Aeromonas salmonicida* subsp. *salmonicida* was isolated from the skin furuncles of juveniles at Farm B that displayed this symptom typical of the chronic form of furunculosis, and also showed hepatic and intestinal petechiae and splenomegaly, all previously associated with infections caused by this pathogen [29]. *Aeromonas hydrophila* was isolated from the fry at Farm C that presented deep cutaneous ulcers with tissue loss at the caudal fin, signs previously reported for infections caused by this bacterium [29]. The high genetic similarity among *Aeromonas* species hampers precise identification by 16S rRNA sequencing and MALDI-TOF MS. Future studies could achieve a higher taxonomic resolution using multilocus sequence analysis (MLSA), whole-genome sequencing (WGS), or other next-generation sequencing (NGS) approaches [22].

*Carnobacterium maltaromaticum* is also a notable pathogen capable of causing ocular lesions and hepatic pathology. It has been isolated from *O. mykiss* [30], *S. trutta* [13], brook trout (*Salvelinus fontinalis*) [31], and other fish species [7]. Clinical signs are generally heterogeneous but overall consistent with septicemia [29]. In this study, *C. maltaromaticum* was isolated on two occasions: at Farm B, from an adult with hepatic and intestinal petechial hemorrhages, and at Farm E, from juveniles exhibiting ocular rupture. Although *P. shigelloides* and *A. encheleia* are less frequently reported as primary pathogens, they may cause disease under specific stress conditions or act opportunistically. *Hafnia alvei* is an opportunistic pathogen that can cause mortality in brown trout; reported clinical signs include melanosis, erratic swimming, and generalized hemorrhagic septicemia [29]. In this study, this bacterium was isolated on three occasions: at Farm A, from fry exhibiting erratic swimming, external petechial hemorrhages, and hepatic hemorrhages, and twice during a visit to Farm E from different cohorts, although only one cohort of juveniles showed fin hemorrhages and splenomegaly, signs previously described for infections caused by this bacterium. *Pseudomonas fulva*, *K. intermedia*, *E. faecium*, and *L. raffinolactis* are not typically implicated as primary etiological agents for the clinical signs observed but may be recovered as opportunistic or secondary invaders, particularly in fish with compromised health [31,32,33,34,35,36]. Additional studies, including infections in experimental fish models and histopathological analyses, are required to determine the actual role of these isolated bacteria in disease outbreaks [7].

This study adopts a field-based approach and was conducted using diagnostic tools commonly available in veterinary laboratories, which must deliver rapid results to enable timely identification and implementation of appropriate treatments for disease management in aquaculture. Although these methods do not always achieve a species-level resolution, they allow for the detection of dominant bacterial taxa during mortality events and support informed therapeutic decisions. Integrating multiple methods, including ITS-1 sequencing and qPCR, substantially increased the confidence in bacterial identification in this study. Future efforts to standardize higher-resolution genetic markers in veterinary diagnostics will be crucial to improving pathogen identification.

Several mortality outbreaks coincide with environmental stressors, such as abrupt increases in water temperature or episodes of high turbidity. In the latter case, turbidity was mainly driven by suspended organic matter (mud and leaf litter), often following heavy rainfall or water releases from upstream hydroelectric plants. These observations support the hypothesis that environmental stress, whether thermal fluctuations or elevated turbidity, can compromise fish immune defenses and favor opportunistic bacterial infections, underscoring the need to monitor and control these physico-chemical water parameters to prevent such stressors [8,9,32]. In the context of climate change, which is predicted to impose more extreme conditions on Mediterranean rivers [2], *S. trutta* populations may become increasingly susceptible to disease.

None of the isolates in this study met the criteria for multidrug resistance (MDR), as defined by European organizations. Resistant bacteria were detected in farms that had not used antibiotics, whereas no resistant pathogenic isolates to florfenicol were found in farms that routinely administer this antibiotic to treat bacterial infections. This pattern suggests that AMR in aquaculture may be driven by environmental factors, water contamination, or the introduction of resistant bacteria from external sources, rather than solely by on-farm antibiotic use. The presence of resistant strains in antibiotic-free farms highlights the complexity of AMR dissemination in aquatic environments and underscores the need for surveillance programs to monitor resistance trends [10].

Interestingly, several of the bacterial species identified, such as *K. intermedia*, *E. faecium*, and *Lactococcus* sp., are not traditionally regarded as primary fish pathogens. Nevertheless, their presence in diseased fish, particularly those exhibiting antimicrobial resistance, suggests a potential role as indicators of resistance dissemination in aquatic environments. Given that AMR surveillance is an increasing priority in aquaculture and public health, further studies should assess whether these bacteria could serve as sentinel organisms for tracking antimicrobial resistance trends in fish-farming systems [12].

Regarding the potential link between resistant bacteria and the degree of upstream human activity, farms located in basins with larger human populations (>2000 inhabitants)—Farms C and E—showed both a higher diversity and frequency of resistant isolates (*A. hydrophila*, *K. intermedia*, *E. faecium*, and *Lactococcus* sp.). Conversely, Farm D, which lacks substantial upstream human settlements, yielded no resistant isolates. Although the sample size was necessarily constrained by field conditions and resources and the approach is primarily descriptive, these findings support the possibility that upstream human activities may introduce or amplify resistant strains (e.g., via wastewater discharge or agricultural runoff). Further research is needed to clarify the relative contributions of these anthropogenic factors.

In Spain, at the time of writing, only three antibiotics are authorized for use in aquatic species such as freshwater salmonids, and all must be prescribed by licensed veterinarians. These agents are marketed as medicated premixes for incorporation into feed and include: (i) A product containing florfenicol as the active ingredient, indicated for rainbow trout to treat furunculosis caused by *A. salmonicida*; (ii) a flumequine-based product, indicated for trout, likewise for furunculosis due to *A. salmonicida*; and (iii) two products from different commercial brands containing oxytetracycline hydrochloride, indicated for eels, carp, gilthead seabream, seabass, turbot, and salmonids to treat infections caused by *A. hydrophila*, *L. garvieae*, and *Vibrio anguillarum*.

For the bacteria detected in this study, there are therapeutic options available that could effectively control the infection. Based on the antibiogram results, the most appropriate antibiotic should be selected, taking into account its authorized use in the target species, the bacterial species involved, and the European Medicines Agency (EMA) classification of the antimicrobial agent. Based on the EMA categorization, and considering the antibiotics available for salmonids, the first-line treatment would be oxytetracycline (Category D, to be used with prudence). Florfenicol, which is in EMA Category C, and whose use should be applied with caution when no antimicrobials in Category D are clinically effective, is particularly effective against Gram-negative bacteria, and, finally, flumequine, which is categorized by the EMA as Category B, whose use is restricted in animals to mitigate the risk to public health.

In the case of outbreaks detected in yolk-sac fry that are not yet feeding independently, no authorized antibiotic bath treatments are currently available in Spain. At present, no parenteral antibiotics are licensed for use in trout. Intramuscular administration of an agent such as florfenicol could provide an individualized treatment route, exerting far less overall selective pressure for antimicrobial resistance (AMR). This targeted approach would be particularly advantageous for treating only clinically affected broodstock in *S. trutta* farms, where production units are smaller and more compartmentalized than in most other aquaculture operations.

Preventive strategies remain the most effective means of mitigating outbreaks and limiting the selection of resistant bacteria in aquaculture. Spain’s National Plan Against Antibiotic Resistance highlights the need to implement strict biosecurity protocols, best aquaculture practices, targeted genetic selection, and the use of functional feed additives such as probiotics, prebiotics, or postbiotics. A preventive approach to disease management should take precedence over antibiotic-based treatments. At the time of this study, no commercial vaccines against *A. salmonicida* were available for trout in Spain; however, autogenous vaccines prepared from the farm’s own bacterial isolates could represent a viable option. In addition, regular monitoring programs focused on early pathogen detection and AMR surveillance could improve fish-health outcomes.

## 5. Conclusions

This study identified Gram-negative bacteria as the predominant group associated with *S. trutta* mortality across five fish farms, with *Aeromonas* spp. consistently linked to outbreaks. Although some antibiotic-resistant bacteria were detected, none met the criteria for multidrug resistance (MDR), and most remained susceptible to at least one of the three antibiotics authorized for fish treatment in Spain.

The detection of resistant bacteria in farms both using and not using antibiotics highlights the complexity of AMR dissemination in aquaculture. This underscores the need for comprehensive surveillance programs that integrate environmental monitoring, prudent antibiotic use, and preventive strategies such as vaccination and functional feed additives aimed at strengthening the microbiota.

These findings reinforce the importance of a One Health approach, recognizing the interconnections between aquaculture, environmental health, and public health. The presence of resistant bacteria in antibiotic-free farms suggests that there are potential external sources of AMR—such as water contamination or wildlife vectors. Collaborative efforts among aquaculture managers, veterinarians, and public health authorities are essential for sustainable disease control.

Additionally, the absence of resistant pathogenic isolates in farms administering florfenicol suggests that this antibiotic remains effective; however, sustained monitoring is necessary to prevent the emergence of resistance. The detection of bacteria such as *K. intermedia*, *E. faecium*, and *Lactococcus* sp. indicates their potential utility as indicators of AMR dissemination and warrants further investigation.

In conclusion, this study provides valuable insights into bacterial isolates, antibiotic resistance, and environmental drivers of *S. trutta* mortality. Enhancing early pathogen detection, promoting responsible antibiotic use, and implementing robust environmental monitoring will strengthen fish-health management, fostering more resilient and sustainable aquaculture systems in the face of climate change and emerging disease threats.

## Figures and Tables

**Table 2:** Bacterial species strains used for the phylogenetic analysis and their Genbank accession number.

Species/Strain	Accession No.	Nucleotide Position
*Aeromonas allosaccharophila* strain CECT 4199	NR_025945.2	79–1435
*Aeromonas bestiarum* strain CIP 74.30	NR_026089.2	79–1435
*Aeromonas bivalvium* strain 868E	NR_043885.1	80–1436
*Aeromonas encheleia* strain CECT 4342	NR_118042.1	79–1435
*Aeromonas eucrenophila* strain NCIMB 74	NR_118946.1	79–1435
*Aeromonas hydrophila* strain ATCC 7966	NR_074841.1	80–1436
*Aeromonas molluscorum* strain LMG 22214	NR_115351.1	64–1420
*Aeromonas rivuli* strain DSM 22539	NR_116880.1	79–1435
*Aeromonas salmonicida* strain CECT 894	NR_043324.1	64–1420
*Aeromonas salmonicida* subsp. *achromogenes* strain 6263/4/5	NR_037011.1	79–1434
*Aeromonas salmonicida* subsp. *masoucida* strain NBRC 13784	NR_113635.1	60–1416
*Aeromonas sobria* strain ATCC 43979	NR_119044.1	85–1441
*Aeromonas veronii bv. veronii* strain ATCC 35624	NR_118947.1	79–1435
*Carnobacterium divergens* strain NBRC 15683	NR_113798.1	60–1426
*Carnobacterium gallinarum* strain DSM 4847	NR_042093.1	82–1444
*Carnobacterium inhibens* subsp. *inhibens* DSM 13024 strain K1	NR_036895.1	82–1450
*Carnobacterium maltaromaticum* strain DSM 20342	NR_044710.2	87–1452
*Enterococcus durans* strain 98D	NR_036922.1	74–1442
*Enterococcus faecium* strain DSM 20477	NR_114742.1	71–1439
*Enterococcus hirae ATCC 9790*	NR_075022.1	85–1453
*Enterococcus lactis* strain BT159	NR_117562.1	40–1408
*Enterococcus pseudoavium* strain LMG 11426	NR_114785.2	81–1445
*Hafnia alvei* strain JCM 1666	NR_112985.1	67–1422
*Hafnia paralvei* strain ATCC 29927	NR_116898.1	59–1414
*Huaxiibacter chinensis* strain 155047	NR_184601.1	82–1435
*Klebsiella aerogenes* strain KCTC 2190	NR_102493.2	85–1438
*Kluyvera ascorbata* strain ATCC 33433	NR_028677.1	35–1388
*Kluyvera intermedia* strain NBRC 102594	NR_114153.1	58–1411
*Lactococcus carnosus* strain TMW 2.1612	NR_174340.1	75–1443
*Lactococcus paracarnosus* strain TMW 2.1615	NR_174341.1	75–1443
*Lactococcus piscium* strain CCUG 32732	NR_043739.1	1–1369
*Lactococcus raffinolactis* strain NBRC 100932	NR_113959.1	62–1427
*Piscirickettsia salmonis* strain LF-89	NR_025980.1	78–1433
*Plesiomonas shigelloides* strain NCIMB 9242	NR_044827.1	78–1431
*Pseudomonas ceruminis* strain BML-PP028	NR_181196.1	79–1431
*Pseudomonas entomophila* strain L48	NR_102854.1	76–1428
*Pseudomonas fulva* strain IAM1529	NR_115610.1	74–1425
*Pseudomonas juntendi* strain BML3	NR_180497.1	79–1431
*Pseudomonas monteilii* strain CIP 104883	NR_024910.1	67–1419
*Pseudomonas parafulva* NBRC 16636 = DSM 17004	NR_040859.1	68–1420
*Pseudomonas* sp. URMO17WK12 I11	LN865164.1	1,267,100–1,268,453
*Serratia inhibens* strain S40	NR_180863.1	83–1437
*Serratia liquefaciens* strain ATCC 27592	NR_122057.1	87–1442

**Table 3:** Integrated 16S rRNA, ITS-1, and MALDI-TOF MS identification of bacterial isolates.

Isolate	qPCR Results	MALDI-TOF MS (Score)	16S rRNA (Id. BLAST)	ITS-1 (Id. BLAST)	Isolate Identity	Genbank Accession No	Size
St-Pi3-L-87	Negative	ND (<1.70)	*P. shigelloides* (97.30%)	*P. shigelloides* (92.48%)	*Plesiomonas* sp.	PQ671489.1	2102 nt 16SrRNA + ITS-1
St-Pi3-VL-99	Not tested	*Aeromonas sobria* (1.99)	*A. salmonicida* (100%)	*A. salmonicida* (99.83%)	*Aeromonas* sp.	PQ671490.1	1423 nt 16SrRNA
St-Pi3-OP-104	Not tested	*Hafnia alvei* (2.03)	*Hafnia alvei* (100%)	*Hafnia alvei* (99.43%)	*Hafnia alvei*	PQ671491.1	1392 nt 16SrRNA
St-Pi5-Sp-107	Not tested	*Aeromonas encheleia* (1.99)	*Aeromonas encheleia* (99.93%)	*Aeromonas encheleia* (100%)	*Aeromonas encheleia*	PQ671492.1	1538 nt 16SrRNA
St-Pi5-L-92	(+) *Carnobacterium maltaromaticum*	*C. maltaromaticum* (2.41)	*C. maltaromaticum* (100%)	*C. maltaromaticum* (99.41%)	*Carnobacterium maltaromaticum*	PQ671493.1	2384 nt 16SrRNA + ITS-1
St-Pi5-Sk-98	Negative	*Pseudomonas fulva* (2.22)	*Pseudomonas fulva* (100%)	*Pseudomonas fulva* (99.62%)	*Pseudomonas fulva*	PQ671494.1	2162 nt 16SrRNA + ITS-1
St-Pi5-Sk-89	(+) *A. salmonicida* subsp. *salmonicida*	*Aeromonas bestiarum* (1.72)	*Aeromonas salmonicida* (100%)	*Aeromonas bestiarum* (97.36%)	*A. salmonicida* subsp. *salmonicida*	PQ671495.1	2437 nt 16SrRNA + ITS-1
St-Pi6-OP-110	Negative	*Aeromonas bestiarum* (1.76)	*Aeromonas salmonicida* (100%)	*Aeromonas bestiarum* (99.83%)	*Aeromonas* sp.	PQ671496.1	1376 nt 16SrRNA
St-Pi6-OP-111	Negative	*Aeromonas hydrophila* (2.01)	*Aeromonas hydrophila* (100%)	*Aeromonas hydrophila* (99.83%)	*Aeromonas hydrophila*	PQ671497.1	1528 nt 16SrRNA
St-Pi6-OP-113	Negative	*Kluyvera intermedia* (2.06)	*Kluyvera intermedia* (100%)	*Kluyvera intermedia* (98.31%)	*Kluyvera intermedia*	PQ671498.1	2274 nt 16SrRNA + ITS-1
St-Pi6-OP-105	Not tested	*Kluyvera intermedia* (1.74)	*Kluyvera intermedia* (99.93%)	*Kluyvera intermedia* (99.08%)	*Kluyvera intermedia*	PQ671499.1	1397 nt 16SrRNA
St-Pi2-Sp-101	Not tested	*Aeromonas eucrenophila* (2.05)	*A. salmonicida* (99.86%)	*Aeromonas bestiarum* (98.03%)	*Aeromonas* sp.	PQ671500.1	1419 nt 16SrRNA
St-Pi7-OP-96	Negative	*Enterococcus faecium* (2.12)	*Enterococcus faecium* (100%)	*Enterococcus faecium* (100%)	*Enterococcus faecium*	PQ671501.1	2337 nt 16SrRNA + ITS-1
St-Pi7-OP-97	Negative	*Aeromonas bestiarum* (1.92)	*Aeromonas sobria* (100%)	*A. salmonicida* (96.79%)	*Aeromonas* sp.	PQ671502.1	2378 nt 16SrRNA + ITS-1
St-Pi7-OP-114	Negative	*Lactococcus raffinolactis* (1.71)	*Lactococcus raffinolactis* (100%)	*Lactococcus raffinolactis* (99.18%)	*Lactococcus raffinolactis*	PQ671503.1	2312 nt 16SrRNA + ITS-1
St-Pi7-OP-115	Negative	*Hafnia alvei* (2.05)	*Hafnia alvei* (100%)	*Hafnia alvei* (98.59%)	*Hafnia alvei*	PQ671504.1	2375 nt 16SrRNA + ITS-1
St-Pi7-OP-116	Negative	*Hafnia alvei* (2.15)	*Hafnia alvei* (100%)	*Hafnia alvei* (98.55%)	*Hafnia alvei*	PQ671505.1	2358 nt 16SrRNA + ITS-1
St-Pi7-OP-118	(+) *Carnobacterium maltaromaticum*	Not tested	*C. maltaromaticum* (99.93%)	*C. maltaromaticum* (99.18%)	*Carnobacterium maltaromaticum*	PQ671506.1	2343 nt 16SrRNA + ITS-1
St-Pi7-OP-119	Negative	*Aeromonas bestiarum* (1.83)	*Aeromonas salmonicida* (100%)	*Aeromonas bestiarum* (96.58%)	*Aeromonas* sp.	PQ671507.1	2408 nt 16SrRNA + ITS-1

**Table 4:** Inhibition zones and interpretation of antibiotic susceptibility test for isolates.

Isolate	Amphenicols	Quinolones	Tetracyclines		Macrolides	Cephalosporins	Aminoglycosides	Penicillin	Combination Agents
	Florfenicol (30 µg)	Flumequine (30 µg)	Oxytetracycline (30 µg)	Doxycycline (30 µg)	Erythromycin (15 µg)	Ceftriaxone (30 µg)	Streptomycin (10 µg)	Penicillin G (10 Units)	Amoxicillin/Clavulanic Acid (30 µg)
St-Pi3-L-87 *Plesiomonas* sp.	36.7 ± 1.2	39.0 ± 0.0	33.0 ± 0.0	32.0 ± 0.8	19.3 ± 0.9	37.3 ± 0.5	10.7 ± 0.5	8.3 ± 0.5	29.0 ± 0.8
	^a^ S	^a^ S	^a^ S	^b^ S	^b^ R_i_	^b^ S	^b^ **R**	^b^ R_i_	^b^ S
St-Pi3-VL-99 *Aeromonas* sp.	33.7 ± 0.5	39.0 ± 1.6	29.0 ± 0.0	26.3 ± 0.5	18.7 ± 0.5	36.7 ± 0.5	8.7 ± 0.5	0.0 ± 0.0	15.0 ± 0.8
	^a^ S ^c (**WT**)^	^a^ S	^c^ S ^c (**WT**)^	^a^ S	^a^ ND ^c (**WT**)^	^d^ S	^a^ ND	^d^ R_i_	^d^ R_i_
St-Pi3-OP-104 *Hafnia alvei*	16.7 ± 1.2	35.3 ± 0.5	21.0 ± 0.8	14.3 ± 0.5	0.0 ± 0.0	25.3 ± 0.9	13.7 ± 0.5	0.0 ± 0.0	8.0 ± 0.0
	^a^ ND	^a^ S	^a^ ND	^b^ S	^b^ R_i_	^b^ S_x_	^b^ I	^b^ R_i_	^b^ R_i_
St-Pi5-Sp-107 *A. encheleia*	28.7 ± 0.9	37.7 ± 0.5	29.0 ± 0.8	25.0 ± 0.8	12.3 ± 0.5	36.7 ± 0.5	14.3 ± 0.5	0.0 ± 0.0	14.7 ± 0.5
	^a^ S	^a^ S	^a^ S	^a^ S	^a^ ND	^d^ S	^a^ ND	^d^ R_i_	^d^ R_i_
St-Pi5-L-92 *C. maltaromaticum*	34.0 ± 0.8	14.0 ± 0.8	34.7 ± 0.9	35.3 ± 0.5	33.3 ± 0.5	0.0 ± 0.0	0.0 ± 0.0	22.0 ± 0.8	30.0 ± 1.6
	^a^ S	^a^ ND	^a^ S	^e^ S	^e^ S	^e^ R_i_	^e^ R_i_	^e^ S	^a^ S
St-Pi5-Sk-98 *Pseudomonas fulva*	34.0 ± 0.8	39.7 ± 0.5	32.0 ± 0.8	28.0 ± 0.8	16.0 ± 0.8	36.0 ± 0.8	14.7 ± 0.5	0.0 ± 0.0	12.0 ± 0.8
	^a^ S	^a^ S	^a^ S	^a^ S	^a^ ND	^a^ S	^a^ ND	^a^ **R**	^a^ ND
St-Pi5-Sk-89 *A. salmonicida* subsp. *salmonicida*	34.0 ± 0.8	39.3 ± 0.5	31.7 ± 0.5	28.0 ± 0.0	16.3 ± 0.5	35.3 ± 0.5	14.7 ± 0.5	0.0 ± 0.0	12.7 ± 0.5
	^a^ S ^c (**WT**)^	^a^ S	^c^ S ^c (**WT**)^	^a^ S	^a^ ND ^c (**WT**)^	^d^ S	^a^ ND	^d^ R_i_	^d^ R_i_
St-Pi6-OP-110 *Aeromonas* sp.	33.7 ± 0.5	44.3 ± 0.5	30.3 ± 0.5	28.0 ± 0.8	18.0 ± 0.0	34.0 ± 0.0	14.3 ± 0.5	0.0 ± 0.0	15.0 ± 0.8
	^a^ S ^c (**WT**)^	^a^ S	^c^ S ^c (**WT**)^	^a^ S	^a^ ND ^c (**WT**)^	^d^ S	^a^ ND	^d^ R_i_	^d^ R_i_
St-Pi6-OP-111 *A. hydrophila*	30.3 ± 0.9	13.7 ± 0.5	9.7 ± 0.5	15.7 ± 0.5	0.0 ± 0.0	34.3 ± 0.5	17.0 ± 0.0	0.0 ± 0.0	13.7 ± 0.5
	^a^ S ^f (**WT**)^	^a^ ND	^a^ **R** ^f (**NWT**)^	^a^ ND	^a^ **R**	^d^ S	^a^ ND	^d^ R_i_	^d^ R_i_
St-Pi6-OP-113 *K. intermedia*	18.7 ± 0.5	29.0 ± 0.8	24.3 ± 0.5	20.7 ± 0.5	0.0 ± 0.0	33.7 ± 0.5	14.7 ± 0.9	0.0 ± 0.0	13.3 ± 1.2
	^a^ ND	^a^ S	^a^ ND	^b^ S	^b^ R_i_	^b^ S	^b^ I	^b^ R_i_	^b^ **R**
St-Pi6-OP-105 *K. intermedia*	18.0 ± 0.0	22.3 ± 0.9	19.0 ± 0.8	16.3 ± 1.2	0.0 ± 0.0	27.3 ± 1.7	12.7 ± 0.5	0.0 ± 0.0	13.7 ± 0.5
	^a^ ND	^a^ ND	^a^ ND	^b^ S	^b^ R_i_	^b^ S	^b^ I	^b^ R_i_	^b^ **R**
St-Pi2-Sp-101 *Aeromonas* sp.	31.7 ± 0.5	40.3 ± 0.5	29.7 ± 0.5	27.0 ± 0.8	15.0 ± 0.8	36.0 ± 0.0	15.7 ± 0.9	0.0 ± 0.0	15.3 ± 0.9
	^a^ S	^a^ S	^a^ S	^a^ S	^a^ ND	^d^ S	^a^ ND	^d^ R_i_	^d^ R_i_
St-Pi7-OP-96 *E. faecium*	32.7 ± 0.9	11.3 ± 0.5	0.0 ± 0.0	12.0 ± 0.8	23.3 ± 0.5	12.3 ± 0.5	0.0 ± 0.0	20.7 ± 0.9	27.7 ± 0.5
	^a^ S	^a^ ND	^a^ **R**	^g^ **R**	^g^ S	^g^ R_i_	^g^ R_i_	^g^ S	^a^ S
St-Pi7-OP-97 *Aeromonas* sp.	0.0 ± 0.0	21.0 ± 0.8	21.3 ± 0.5	21.7 ± 0.5	0.0 ± 0.0	20.3 ± 0.5	10.3 ± 0.5	0.0 ± 0.0	11.0 ± 0.0
	^a^ **R**	^a^ ND	^a^ ND	^a^ ND	^a^ **R**	^d^ I	^a^ ND	^d^ R_i_	^d^ R_i_
St-Pi7-OP-114 *L. raffinolactis*	35.3 ± 1.2	0.0 ± 0.0	36.0 ± 0.8	36.3 ± 0.9	37.3 ± 0.5	34.0 ± 0.8	16.3 ± 0.5	35.3 ± 1.2	39.7 ± 1.7
	^a^ S	^a^ **R**	^a^ S	^i^ S	^h^ S	^i^ S	^a^ ND	^h^ S	^a^ S
St-Pi7-OP-115 *Hafnia alvei*	16.7 ± 0.5	36.3 ± 0.5	20.0 ± 0.0	15.0 ± 0.8	0.0 ± 0.0	28.0 ± 0.8	14.7 ± 0.5	0.0 ± 0.0	8.7 ± 0.5
	^a^ ND	^a^ S	^a^ ND	^b^ S	^b^ R_i_	^b^ S_x_	^b^ I	^b^ R_i_	^b^ R_i_
St-Pi7-OP-116 *Hafnia alvei*	12.0 ± 0.8	33.3 ± 0.5	18.3 ± 0.9	16.7 ± 0.5	8.0 ± 0.0	28.7 ± 0.9	16.7 ± 0.5	9.3 ± 0.9	18.7 ± 0.5
	^a^ ND	^a^ S	^a^ ND	^b^ S	^b^ R_i_	^b^ S_x_	^b^ S	^b^ R_i_	^b^ R_i_
St-Pi7-OP-118 *C. maltaromaticum*	30.3 ± 2.1	10.3 ± 0.5	32.3 ± 2.1	33.7 ± 0.9	32.0 ± 0.8	0.0 ± 0.0	0.0 ± 0.0	23.0 ± 0.8	31.3 ± 0.9
	^a^ S	^a^ ND	^a^ S	^e^ S	^e^ S	^e^ R_i_	^e^ R_i_	^e^ S	^a^ S
St-Pi7-OP-119 *Aeromonas* sp.	32.0 ± 0.8	36.0 ± 0.0	29.3 ± 0.5	26.0 ± 0.8	15.0 ± 0.8	31.7 ± 0.5	16.0 ± 0.0	0.0 ± 0.0	14.0 ± 0.8
	^a^ S	^a^ S	^a^ S	^a^ S	^a^ ND	^d^ S	^a^ ND	^d^ R_i_	^d^ R_i_

Inhibition-zone diameters are expressed in millimeters (mm). The bacterial strains were classified as susceptible (S), intermediate (I), or resistant (R). For inhibition zones smaller than 25 mm, and in the absence of specific guidelines or references, the results were recorded as not determined (ND). ^a^ No CLSI interpretive criteria are available; results were interpreted as described in Section 2.8 (Materials and Methods). ^b^ Interpreted according to M100 Ed33, disk diffusion method for Enterobacterales. ^c^ Interpreted according to Vet04 Ed3E, disk diffusion method for *A. salmonicida*. ^d^ Interpreted according to M45 Ed3, disk diffusion method for *Aeromonas* spp. ^e^ The M45 Ed3 manual says that for this Gram-positive bacilli, the results can be interpreted according to M100 Ed33, disk diffusion method for *Enterococcus* spp. ^f^ Interpreted according to Vet04 Ed3E, disk diffusion method for *A. hydrophila*. ^g^ Interpreted according to M100 Ed33, disk diffusion method for *Enterococcus* spp. ^h^ The M45 Ed3 manual says that for *Lactococcus* spp. the results against penicillin and erythromycin can be interpreted according to M100 Ed33, disk diffusion method for *Staphylococcus* spp. ^i^ The M45 Ed3 manual says that for *Lactococcus* spp. the results can be interpreted according to M100 Ed33, disk diffusion method for *Streptococcus* spp. *Viridans* Group. When the CBC of an isolate is recorded as R_i_, it indicates that the bacteria are intrinsically resistant to an antibiotic. When the CBC of an isolate is recorded as S_x_, it indicates that the agent may be ineffective within a few days after initiation of therapy. WT, wild type, NWT, no wild type.

**Table 5:** Clinical signs, bacterial isolates and antimicrobial resistance profiles recorded in trout farms during site visits (2022–2023).

Farm	Adjacent and Upstream Population	Visit	Date	Weather Events	Symptoms	Bacterial Isolates Identified	AMR
**A**	c. 1600 inhabitants	1st	Mid-Spring 2022	Water temp. 4 °C above the usual levels	Broodstock exhibiting erratic swimming, ascitic fluid in the peritoneal cavity, hepatomegaly, hepatic hemorrhages, stomach dilation, and gallbladder distension	St-Pi3-L-87 *Plesiomonas* sp. (hepatic tissue)	Streptomycin
		2nd	Mid-Spring 2023	Increase in water temperature	Yolk-sac fry showing melanosis and hemorrhagic lesions around the head and gill regions	St-Pi3-VL-99 *Aeromonas* sp. (sac fry)	None
		3rd	Late Summer 2023	Increase in water temperature	Fry exhibiting erratic swimming, petechial hemorrhages along the lateral line, gill redness, liver hemorrhages, and enlarged posterior kidney	St-Pi3-OP-104 *Hafnia alvei* (organs pool; Pi3.L1)	None
**B**	c. 1600 inhabitants	1st	Mid-Spring 2022	Increase in the turbidity of the river water	Fry exhibiting erratic swimming, ocular hemorrhages, loss of the eyeballs, gill pallor, hemorrhagic skin lesions, hepatomegaly, and gastric dilation with mucous content	St-Pi5-Sp-107 *Aeromonas encheleia* (pooled spleen tissue)	None
		2nd	Mid-Autumn 2022	None	Juveniles (batch Pi5.L1) exhibiting furuncles, petechiae in the liver and intestines, and splenomegaly	St-Pi5-Sk-98 *Pseudomonas fulva* (skin boils and abscesses)	Penicillin G
						St-Pi5-Sk-89 *A. salmonicida* subsp. *salmonicida* (skin boils and abscesses)	None
					Adult (batch Pi5.L2) displaying melanosis, petechial hemorrhages in liver and intestines	St-Pi5-L-92 *Carnobacterium maltaromaticum* (hepatic tissue)	None
**C**	c. 7300 inhabitants	1st	Mid-Summer 2023	None	Fry (batch Pi6.L1) exhibiting deep cutaneous ulcers, loss of muscle tissue, erosion and disappearance of the caudal fin	St-Pi6-OP-110 *Aeromonas* sp. (pooled tissue samples of liver and cutaneous lesions)	None
						St-Pi6-OP-111 *Aeromonas hydrophila* (pooled tissue samples of liver and cutaneous lesions)	Oxytetracycline Erythromycin
					Juveniles (batch Pi6.L2) exhibiting hepatomegaly and splenomegaly	St-Pi6-OP-113 *Kluyvera intermedia* (pooled tissue samples of liver, kidney, spleen, and ocular lesions)	Amoxicillin/clav. acid
		2nd	Mid-Autumn 2023	Heavy rainfall event increased the turbidity of the incoming water	Juveniles exhibited rupture of one or both eyeballs, hepatomegaly, and liver discoloration	St-Pi6-OP-105 *Kluyvera intermedia* (pooled tissue samples of liver, kidney, spleen, and muscle)	Amoxicillin/clav. acid
**D**	c. 100 inhabitants	1st	Early Autumn 2022	None	Juveniles exhibiting melanosis; no internal lesions were detected	St-Pi2-Sp-101 *Aeromonas* sp. (pooled tissue from liver and spleen)	None
**E**	c. 2100 inhabitants	1st	Mid-Summer 2023	None	Juveniles (batch Pi7.L1; size 9–10 cm) exhibiting cutaneous wounds with loss of muscle or fin tissue but no internal organ lesions	St-Pi7-OP-96 *Enterococcus faecium* (pooled tissue samples of liver, spleen, kidney, and wounded skin)	Oxytetracycline Doxycycline
						St-Pi7-OP-115 *Hafnia alvei* (pooled tissue samples of liver, spleen, kidney, and wounded skin)	None
						St-Pi7-OP-114 *Lactococcus raffinolactis* (pooled tissue samples of liver, spleen, kidney, and wounded skin)	Flumequine
					Juveniles (batch Pi7.L2; size 12–16 cm) exhibiting dorsal fin hemorrhages and gill petechiae, distended gallbladder, and abundant mucous gastric contents	St-Pi7-OP-97 *Aeromonas* sp. (pooled tissue samples of wounded skin, kidney, liver, and spleen)	Florfenicol Erythromycin
					Juveniles (batch Pi7.L3; size 17–18 cm) exhibiting fin hemorrhages and splenomegaly	St-Pi7-OP-116 *Hafnia alvei* (pooled tissue samples of liver, kidney, and spleen)	None
		2nd	Late Autumn 2023	None	Juveniles exhibiting ruptured eyeballs, erosion of the caudal fin, and hepatomegaly with hepatic hemorrhages	St-Pi7-OP-118 *Carnobacterium malta-romaticum* (pooled tissue samples of liver and kidney)	None
						St-Pi7-OP-119 *Aeromonas* sp. (pooled tissue samples of liver and kidney)	None

## Data Availability

This study has contributed 16S rRNA or contigs of 16S rRNA + ITS-1 sequences from various bacteria to the GenBank database. These sequences, used in constructing a phylogenetic tree along with reference sequences from GenBank, are publicly available for consultation and use in research. The specific details and GenBank accession numbers for these sequences can be found in the Section 3 of this article.

## References

[1] García-Vega A., Fuentes-Pérez J.F., Leunda Urretabizkaia P.M., Ardaiz Ganuza J., Sanz-Ronda F.J. (2022). Upstream migration of anadromous and potamodromous brown trout: Patterns and triggers in a 25-year overview. Hydrobiologia.

[2] Vera M., Aparicio E., Heras S., Abras A., Casanova A., Roldán M.I., García-Marin J.-L. (2023). Regional environmental and climatic concerns on preserving native gene pools of a least concern species: Brown trout lineages in Mediterranean streams. Sci. Total Environ..

[3] World Organisation for Animal Health (WOAH) (2024). Diseases of Fish. Manual of Diagnostic Tests for Aquatic Animals.

[4] European Parliament and Council of the European Union (2016). Regulation (EU) 2016/429 of the European Parliament and of the Council of 9 March 2016 on Transmissible Animal Diseases and Amending and Repealing Certain Acts in the Area of Animal Health (“Animal Health Law”).

[5] Rathinam R.B., Iburahim S.A., Ramanan S.S., Tripathi G. (2022). A scientometric mapping of research on Aeromonas infection in fish across the world (1998–2020). Aquac. Int..

[6] Revina O., Avsejenko J., Cīrule D., Valdovska A. Antimicrobial Resistance of Aeromonas spp. Isolated from the Sea Trout (*Salmo trutta* L.) in Latvia. 2017; pp. 271–275. http://llufb.llu.lv/conference/Research-for-Rural-Development/2017/LatviaResRuralDev_23rd_2017_vol1-271-275.pdf.

[7] Austin B., Austin D.A. (2016). Bacterial Fish Pathogens.

[8] Elgendy M.Y., Ali S.E., Abbas W.T., Algammal A.M., Abdelsalam M. (2023). The role of marine pollution on the emergence of fish bacterial diseases. Chemosphere.

[9] Uren Webster T.M., Consuegra S., Garcia De Leaniz C. (2021). Early life stress causes persistent impacts on the microbiome of Atlantic salmon. Comp. Biochem. Physiol. Part D Genom. Proteom..

[10] Milijasevic M., Veskovic-Moracanin S., Babic Milijasevic J., Petrovic J., Nastasijevic I. (2024). Antimicrobial Resistance in Aquaculture: Risk Mitigation within the One Health Context. Foods.

[11] Aerts M., Baron S., Bortolaia V., Hendriksen R., Guerra B., Stoicescu A., Beloeil P. (2024). Technical specifications for a EU-wide baseline survey of antimicrobial resistance in bacteria from aquaculture animals. EFSA J..

[12] Marti E., Huerta B., Rodríguez-Mozaz S., Barceló D., Marcé R., Balcázar J.L. (2018). Abundance of antibiotic resistance genes and bacterial community composition in wild freshwater fish species. Chemosphere.

[13] Vargas-González A., Barajas M., Pérez-Sánchez T. (2024). Isolation of Lactic Acid Bacteria (LAB) from salmonids for potential use as probiotics: In vitro assays and toxicity assessment of *Salmo trutta* embryonated eggs. Animals.

[14] Mohsina K., Kaur M., Bowman J.P., Powell S., Tamplin M.L. (2020). qPCR quantification of *Carnobacterium maltaromaticum*, *Brochothrix thermosphacta*, and *Serratia liquefaciens* growth kinetics in mixed culture. J. Microbiol. Methods.

[15] Torres-Corral Y., Fernández-Álvarez C., Santos Y. (2019). High-throughput identification and quantification of *Vagococcus salmoninarum* by SYBR Green I-based real-time PCR combined with melting curve analysis. J. Fish Dis..

[16] Balcázar J.L., Vendrell D., De Blas I., Ruiz-Zarzuela I., Gironés O., Múzquiz J.L. (2007). Quantitative detection of *Aeromonas salmonicida* in fish tissue by real-time PCR using self-quenched, fluorogenic primers. J. Med. Microbiol..

[17] Keeling S.E., Johnston C., Wallis R., Brosnahan C.L., Gudkovs N., McDonald W.L. (2012). Development and validation of real-time PCR for the detection of *Yersinia ruckeri*: *Yersinia ruckeri* real-time PCR. J. Fish Dis..

[18] Sabat A.J., van Zanten E., Akkerboom V., Wisselink G., van Slochteren K., de Boer R.F., Hendrix R., Friedrich A.W., Rossen J.W.A., Kooistra-Smid A.M.D.M. (2017). Targeted next-generation sequencing of the 16S-23S rRNA region for culture-independent bacterial identification—Increased discrimination of closely related species. Sci. Rep..

[19] Janda J.M., Abbott S.L. (2007). 16S rRNA Gene Sequencing for Bacterial Identification in the Diagnostic Laboratory: Pluses, Perils, and Pitfalls. J. Clin. Microbiol..

[20] Weisburg W.G., Barns S.M., Pelletier D.A., Lane D.J. (1991). 16S ribosomal DNA amplification for phylogenetic study. J. Bacteriol..

[21] Çağatay İ.T. (2024). Use of proteomic-based MALDI-TOF mass spectra for identification of bacterial pathogens in aquaculture: A review. Aquac. Int..

[22] MacAulay S., Ellison A.R., Kille P., Cable J. (2022). Moving towards improved surveillance and earlier diagnosis of aquatic pathogens: From traditional methods to emerging technologies. Rev. Aquac..

[23] Kumar S., Stecher G., Tamura K. (2016). MEGA7: Molecular Evolutionary Genetics Analysis Version 7.0 for Bigger Datasets. Mol. Biol. Evol..

[24] Nei M., Kumar S. (2000). Molecular Evolution and Phylogenetics.

[25] Lewis J.S., Weinstein M.P., Bobenchik A.M., Campeau S., Cullen S.K., Dingle T., Galas M.F., Humphries R.M., Krin T.J., Limbago B. (2023). M100 Performance Standards for Antimicrobial Susceptibility Testing.

[26] Hindler J.A., Humphries R.M., Richter S.S., Jorgensen J.H., Bernard K., Killian S.B., Bodeis-Jones S., Kohner P., Castanheira M., Matuschek E. (2016). M45 Methods for Antimicrobial Dilution and Disk Susceptibility Testing of Infrequently Isolated or Fastidious Bacteria.

[27] Miller R.A., Gieseker C.M., Avendaño-Herrera R., Baron S., Buller N., Burbick C.R., Chuanchuen R., Dalsgaard I., Declercq A.M. (2020). VET04 Performance Standards for Antimicrobial Susceptibility Testing of Bacteria Isolated from Aquatic Animals.

[28] Magiorakos A.P., Srinivasan A., Carey R.B., Carmeli Y., Falagas M.E., Giske C.G., Harbarth S., Hindler J.F., Kahlmeter G., Olsson-Liljequist B. (2012). Multidrug-resistant, extensively drug-resistant and pandrug-resistant bacteria: An international expert proposal for interim standard definitions for acquired resistance. Clin. Microbiol. Infect..

[29] Noguera P.A. (2013). A Colour Atlas of Salmonid Diseases.

[30] Smith S.A., Newman S.J., Harrison C.E., Loch T.P. (2023). First isolation of *Carnobacterium maltaromaticum* from farmed rainbow trout in Virginia. J. Aquat. Anim. Health.

[31] Pastorino P., Colussi S., Pizzul E., Varello K., Menconi V., Mugetti D., Tomasoni M., Esposito G., Bertoli M., Bozzetta E. (2021). The unusual isolation of *carnobacteria* in eyes of healthy salmonids in high-mountain lakes. Sci. Rep..

[32] Orozova P., Sirakov I., Chikova V., Popova R., Al-Harbi A.H., Crumlish M., Austin B. (2014). Recovery of *Hafnia alvei* from diseased brown trout, *Salmo trutta* L., and healthy noble crayfish, *Astacus astacus* (L.), in Bulgaria. J. Fish Dis..

[33] Duman M., Altun S., Saticioglu I.B., Romalde J.L. (2025). A review of bacterial disease outbreaks in rainbow trout (*Oncorhynchus mykiss*) reported from 2010 to 2022. J. Fish Dis..

[34] Clinton M., Kintner A.H., Delannoy C.M.J., Brierley A.S., Ferrier D.E.K. (2020). Molecular identification of potential aquaculture pathogens adherent to cnidarian zooplankton. Aquaculture.

[35] Farkas A., Butiuc-Keul A., Carpa R., Szekeres E., Teban-Man A., Coman C. (2025). Overlooked *Enterobacterales* as hosts of antimicrobial resistance in aquatic environments. Sci. Rep..

[36] Abdel-Raheem S.M., Khodier S.M., Almathen F., Hanafy A.S.T., Abbas S.M., Al-Shami S.A., Al-Sultan S.I., Alfifi A., El-Tarabili R.M. (2024). Dissemination, virulence characteristic, antibiotic resistance determinants of emerging linezolid and vancomycin-resistant *Enterococcus* spp. in fish and crustacean. Int. J. Food Microbiol..

